# TSLP/dendritic cell axis promotes CD4^+^ T cell tolerance to the gut microbiome

**DOI:** 10.1172/jci.insight.160690

**Published:** 2023-07-10

**Authors:** Jonathan L. Messerschmidt, Marjan Azin, Kaitlin E. Dempsey, Shadmehr Demehri

**Affiliations:** Center for Cancer Immunology and Cutaneous Biology Research Center, Department of Dermatology and Center for Cancer Research, Massachusetts General Hospital and Harvard Medical School, Boston, Massachusetts, USA.

**Keywords:** Gastroenterology, Immunology, Adaptive immunity, Cytokines, Tolerance

## Abstract

Thymic stromal lymphopoietin (TSLP) overexpression is widely associated with atopy. However, TSLP is expressed in normal barrier organs, suggesting a homeostatic function. To determine the function of TSLP in barrier sites, we investigated the impact of endogenous TSLP signaling on the homeostatic expansion of CD4^+^ T cells in adult mice. Surprisingly, incoming CD4^+^ T cells induced lethal colitis in adult *Rag1*-knockout animals that lacked the TSLP receptor (Rag1^KO^Tslpr^KO^). Endogenous TSLP signaling was required for reduced CD4^+^ T cell proliferation, Treg differentiation, and homeostatic cytokine production. CD4^+^ T cell expansion in Rag1^KO^Tslpr^KO^ mice was dependent on the gut microbiome. The lethal colitis was rescued by parabiosis between Rag1^KO^Tslpr^KO^ and Rag1^KO^ animals and wild-type dendritic cells (DCs) suppressed CD4^+^ T cell–induced colitis in Rag1^KO^Tslpr^KO^ mice. A compromised T cell tolerance was noted in Tslpr^KO^ adult colon, which was exacerbated by anti–PD-1 and anti–CTLA-4 therapy. These results reveal a critical peripheral tolerance axis between TSLP and DCs in the colon that blocks CD4^+^ T cell activation against the commensal gut microbiome.

## Graphical abstract

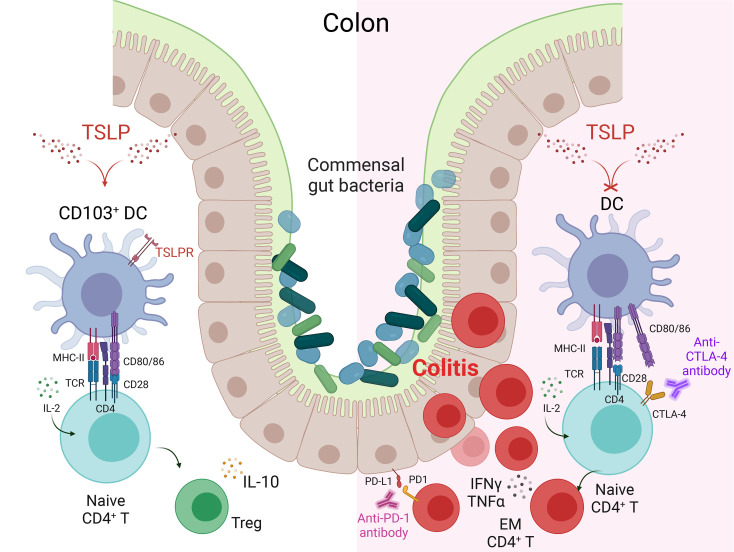

## Introduction

The barrier organs, including the skin, lung, and gastrointestinal (GI) tract, are essential to the initiation of proper immune response to environmental insults. Alarmin cytokines released at barrier sites are known to mediate an initiating alarm signal to tissue-resident and circulating immune cells (1). Among them, the type 2 alarmins, interleukin 25 (IL-25), IL-33, and thymic stromal lymphopoietin (TSLP) skew barrier immune responses toward type 2 immunity, which includes Th2 differentiation, IgE class switching of B cells, and the induction mast cells and eosinophils (2). These cytokines are known to protect the host against helminth and parasitic infections (3). However, IL-25, IL-33, and TSLP have been mostly studied for their role in the induction of allergic inflammation at barrier sites (4) and it is unclear whether these cytokines play any role in other inflammatory conditions that affect barrier organs. Homeostatic T cell expansion is implicated in the maintenance of peripheral T cells and regulation of barrier immunity; however, the precise mechanism underlying homeostatic T cell expansion and the role of type 2 alarmin cytokines in this process are unclear (5, 6). Thus, it is critical to determine the expression and function of type 2 alarmins during the homeostatic T cell proliferation at barrier organs.

Among type 2 alarmin cytokines, TSLP is found to be uniformly expressed under steady-state conditions across the barrier organs (7, 8). However, IL-33 shows differential expression and IL-25 is undetectable in a subset of the barrier organs (9, 10). TSLP interacts with a heterodimeric receptor consisting of the TSLP receptor (TSLPR) chain and the IL-7Rα chain to activate several pathways, including JAK1/2, STAT5, PI3K, and NF-κB (11). Although CD4^+^ T cells are the predominant adaptive immune cell target of TSLP (12), a broad set of adaptive and innate immune cells respond to TSLP, including CD8^+^ T cells and dendritic cells (DCs) (13, 14). In the skin and lung, TSLP is rapidly upregulated following epithelial barrier disruption, leading to the development of atopic dermatitis and asthma, respectively. In the colon, TSLP induction leads to a productive Th2 response that eliminates parasite infection (3, 15) and mediates mucosal healing after exposure to an inflammatory insult (16, 17). Collectively, these studies reveal that TSLP induction has diverse effects on disease states across barrier organs; however, the impact of baseline TSLP expression on the immunological programs of normal barrier organs is uncertain.

Adoptive T cell transfer into recombination-activating gene knockout (*Rag1^–/–^* or Rag1^KO^) and sublethally irradiated wild-type (WT) mice have been used to examine homeostatic CD4^+^ T cell expansion in adult animals (18). Spontaneous CD4^+^ T cell expansion in Rag1^KO^ animals occurs rapidly, with multiple cell divisions per day, and is regulated by antigen presentation on CD11c^+^ DCs (19–21). Homeostatic expansion of T cells in sublethally irradiated WT mice occurs on a longer time scale, with a single cell division every 3–4 days while requiring an antigenic reaction (19, 22). These homeostatic T cell expansion models require the growth-promoting, homeostatic cytokine IL-7 and TCR-MHC engagement to initiate proliferation while needing other cytokine inputs to determine the functional outcome of the resulting T cell expansion (21–24). While Rag1^KO^ and sublethally irradiated mice do not represent a fully intact immune environment, they provide a suitable model to study the effects of alarmin cytokines on the establishment of T cell homeostasis in barrier organs of adult animals.

To determine the role of alarmin cytokines in maintaining tissue-immune homeostasis, we examined IL-25, IL-33, and TSLP levels in barrier organs of healthy adult WT mice. TSLP was the only cytokine uniformly expressed across the barrier sites. Adoptive transfer of WT CD4^+^ T cells into TSLPR-deficient Rag1^KO^ mice (*Rag1^–/–^* *Tslpr^–/–^* or Rag1^KO^Tslpr^KO^) caused fulminant, lethal colitis. This lethality was brought on by microbiome-dependent increases in T cell proliferation and IFN-γ expression, revealing that TSLP was required for peripheral CD4^+^ T cell tolerance to the gut microbiome. Parabiosis and WT CD11c^+^ DC transfer rescued Rag1^KO^Tslpr^KO^ mice from adoptive T cell transfer–induced colitis. Importantly, Tslpr^KO^ mice were prone to colitis following adoptive CD4^+^ T cell transfer and showed heightened expansion of colonic CD4^+^ T cells in response to immune checkpoint blockade (ICB) therapy. Taken together, our findings reveal that TSLP acts as a homeostatic and tolerogenic alarmin in the gut by regulating DC–T cell communication.

## Results

### TSLP signaling prevents lethal colitis during homeostatic CD4^+^ T cell expansion.

**Figure 1:**
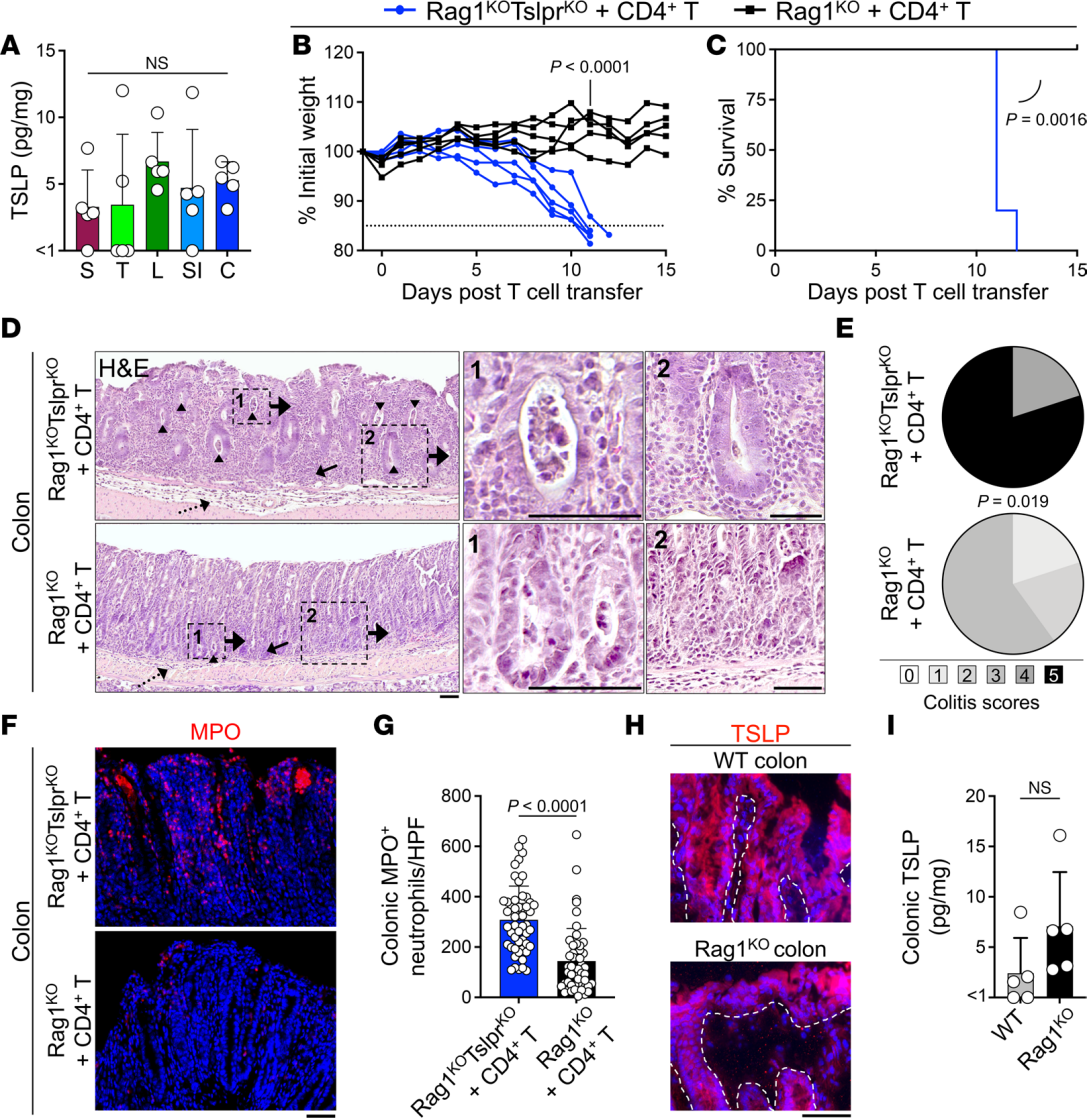
TSLP signaling is required to prevent acute, lethal colitis following adoptive CD4^+^ T cell transfer. (**A**) TSLP protein concentration in WT mice (*n* = 5) skin (S), trachea (T), lung (L), small intestine (SI), and colon (C). Each dot represents 1 mouse. Ordinary 1-way ANOVA. (**B** and **C**) Weight (**B**) and survival (**C**) of Rag1^KO^Tslpr^KO^ (*n* = 5) and Rag1^KO^ (*n* = 5) mice following adoptive transfer of 2 million purified WT CD4^+^CD25^–^ T cells. Weight: unpaired, 2-tailed *t* test; survival: log-rank test. Experimental data were verified in a second independent experiment. (**D**) Representative H&E staining of Rag1^KO^Tslpr^KO^ cells and Rag1^KO^ colon at the moribund state or 15 days after T cell transfer. Colon mucosal (solid arrows) and submucosal (dotted arrows) inflammation and crypt abscess formation (arrowheads) are highlighted. 1, High-power view (×800) of crypt abscess with luminal neutrophilic infiltration. 2, High-power view (×400) of goblet cell loss, cryptitis, and a dense lymphocyte-dominated inflammatory environment in Rag1^KO^Tslpr^KO^ colon and inflammatory environment of Rag1^KO^ colon. (**E**) Colitis scoring of Rag1^KO^Tslpr^KO^ (*n* = 5) and Rag1^KO^ (*n* = 5) mice (Fisher’s exact test). (**F**) Representative myeloperoxidase (MPO) staining of Rag1^KO^Tslpr^KO^ and Rag1^KO^ colons after CD4^+^ T cell transfer. (**G**) Quantification of MPO^+^ neutrophils in Rag1^KO^Tslpr^KO^ (*n* = 5) and Rag1^KO^ (*n* = 5) colons. MPO^+^ cells were quantified in 10 randomly selected high-power fields (HPFs) per colon. Each dot represents 1 HPF. (**H**) Representative immunofluorescent staining of TSLP (red) in WT and Rag1^KO^ colons. Dashed lines highlight the epithelial basement membrane. (**I**) TSLP protein concentration in Rag1^KO^ (*n* = 5) versus WT (*n* = 5) colon. Each dot represents 1 mouse. Scale bars: 50 μm. Bar graphs show mean + SD. Unpaired, 2-tailed *t* test (**G** and **I**). NS, not significant.

We examined IL-25, IL-33, and TSLP expression in the skin, trachea, lung, small intestine, and colon of 8-week-old WT mice on the C57BL/6 background. TSLP was uniformly detectable across the barrier organs, while IL-33 showed variable expression levels and IL-25 was undetectable in a subset of the organs (Figure 1A and Supplemental Figure 1, A and B; supplemental material available online with this article; https://doi.org/10.1172/jci.insight.160690DS1). To determine the role of endogenous TSLP in barrier organs, we examined the homeostatic expansion of CD4^+^ T cells in T and B cell–deficient Rag1^KO^ mice that lacked TSLPR (18). Rag1^KO^Tslpr^KO^ mice rapidly lost weight and reached a moribund state by 12 days after adoptive naive WT CD4^+^CD25^–^ T cell transfer, while Rag1^KO^ showed no appreciable weight loss after T cell transfer (*P* < 0.0001; Figure 1, B and C, and Supplemental Figure 1C). The clinical and histological examination of the barrier and other vital organs revealed an inflammatory reaction in the colon, which was markedly more severe in Rag1^KO^Tslpr^KO^ compared with Rag1^KO^ mice after adoptive T cell transfer (*P* = 0.019; Figure 1, D and E, and Supplemental Figure 1, D–G). Consistently, a more prominent neutrophilic infiltrate was detectable in Rag1^KO^Tslpr^KO^ compared with Rag1^KO^ colon after adoptive T cell transfer (*P* < 0.0001; Figure 1, F and G). Skin, trachea, lung, small intestine, brain, kidney, liver, spleen, pancreas, and peripheral blood did not reveal any signs of inflammation after adoptive T cell transfer (Supplemental Figure 1, F and G). TSLP was expressed by the epithelial lining of WT and Rag1^KO^ colons at similar levels, suggesting that epithelial TSLP secretion in the colon contributed to the observed phenotype (Figure 1, H and I).

### TSLP suppresses pathological CD4^+^ T cell proliferation in the colon.

**Figure 2:**
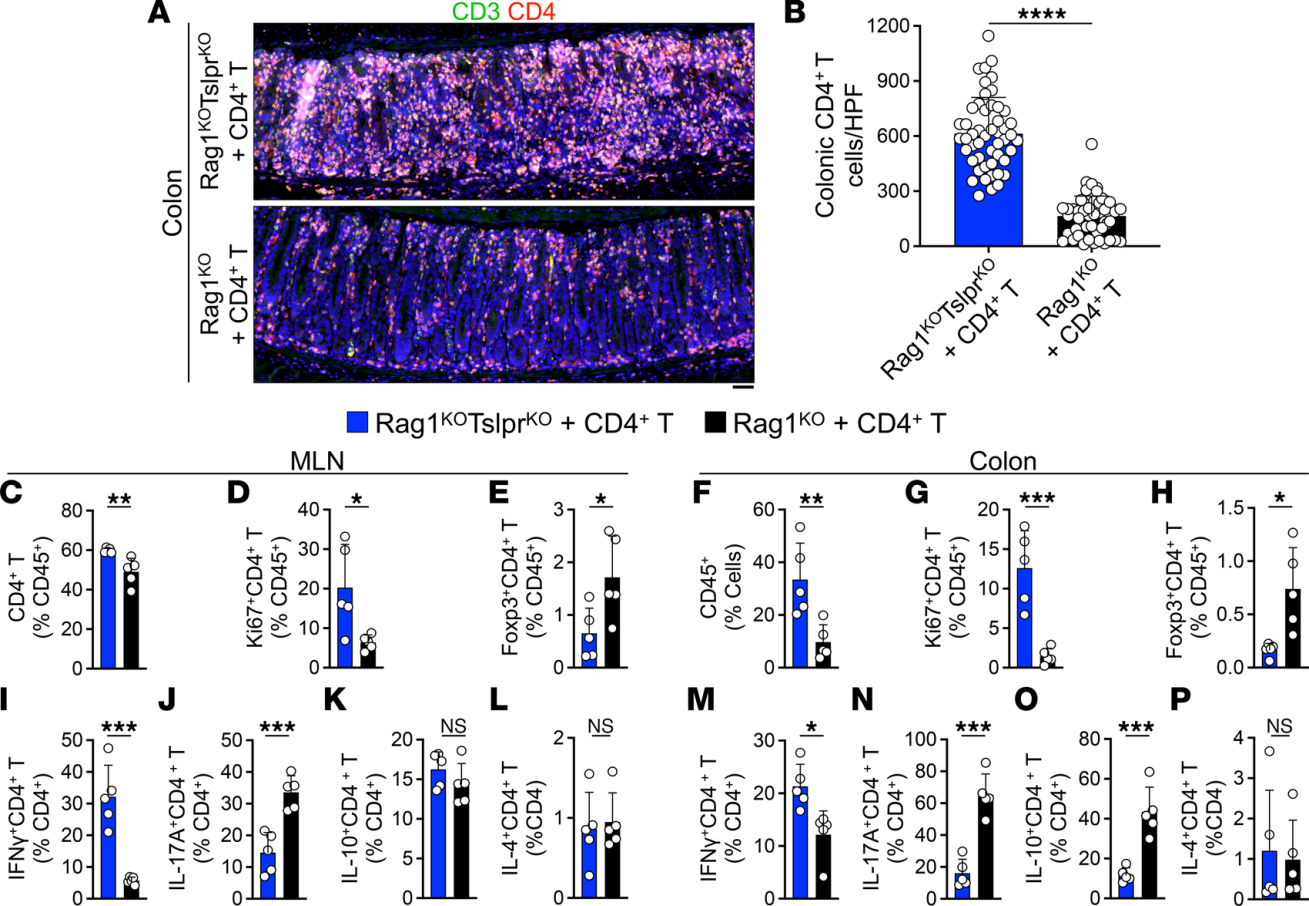
CD4^+^ T cells massively expand in TSLP receptor–deficient colon. (**A**) Representative CD3/CD4 staining of Rag1^KO^Tslpr^KO^ and Rag1^KO^ colons at the moribund state or 15 days after CD4^+^ T cell transfer. Scale bar: 50 μm. (**B**) CD4^+^ T cell counts in Rag1^KO^Tslpr^KO^ (*n* = 5) and Rag1^KO^ (*n* = 5) colons from 1 experiment that was validated in a second independent experiment. CD4^+^CD3^+^ cells were counted in 10 randomly selected high-powered fields (HPFs, overall magnification ×200) per colon. Each dot represents 1 HPF. (**C**–**E**) Flow cytometric quantification of CD4^+^ T (**C**), Ki67^+^CD4^+^ T (**D**), and Foxp3^+^CD4^+^ Treg (**E**) frequency of MLNs from Rag1^KO^Tslpr^KO^ (*n* = 5) and Rag1^KO^ (*n* = 5) mice. Experimental data were verified in a second independent experiment. (**F**–**H**) Flow cytometric quantification of CD4^+^ T (**F**), Ki67^+^CD4^+^ T (**G**), and Foxp3^+^CD4^+^ Treg (**H**) frequency in Rag1^KO^Tslpr^KO^ (*n* = 5) and Rag1^KO^ (*n* = 5) colons. Experimental data were verified in a second independent experiment. (**I**–**L**) Flow cytometric quantification of IFN-γ^+^ (**I**), IL-17A^+^ (**J**), IL-10^+^ (**K**), and IL-4^+^ (**L**) CD4^+^ T cell frequency of MLNs from Rag1^KO^Tslpr^KO^ (*n* = 5) and Rag1^KO^ (*n* = 5) mice. Experimental data were verified in a second independent experiment. (**M**–**P**) Flow cytometric quantification of IFN-γ^+^ (**M**), IL-17A^+^ (**N**), IL-10^+^ (**O**), and IL-4^+^ (**P**) CD4^+^ T cell frequency in Rag1^KO^Tslpr^KO^ (*n* = 5) and Rag1^KO^ (*n* = 5) colons. Experimental data were verified in a second independent experiment. Single-cell suspensions from MLNs and colon were stimulated for 4 hours with PMA/ionomycin + brefeldin A and analyzed for cytokine production. Each dot represents 1 mouse; bar graphs show mean + SD. *P < 0.05, **P < 0.01, ***P < 0.001, ****P < 0.0001 by unpaired, 2-tailed *t* test. NS, not significant.

To determine the cause of lethal colitis in Rag1^KO^Tslpr^KO^ mice, we characterized the adoptively transferred CD4^+^ T cells in the recipient animals. Following the adoptive transfer, Rag1^KO^Tslpr^KO^ mice showed massive expansion of CD4^+^ T cells in the colonic mucosa compared with Rag1^KO^ mice (*P* < 0.0001; Figure 2, A and B). Rag1^KO^Tslpr^KO^ mesenteric lymph nodes (MLNs) showed increased CD4^+^ T cell frequency (*P* = 0.0085, Figure 2C) and a higher percentage of proliferating Ki67^+^CD4^+^ T cells compared with Rag1^KO^ MLNs (*P* = 0.0231, Figure 2D). In addition, Rag1^KO^Tslpr^KO^ MLNs contained fewer Foxp3^+^CD4^+^ Tregs compared with Rag1^KO^ MLNs (*P* = 0.0322, Figure 2E). The pathological inflammation in the Rag1^KO^Tslpr^KO^ colon was characterized by a marked increase in total CD45^+^ leukocyte frequency (*P* = 0.0085, Figure 2F) and a higher percentage of Ki67^+^CD4^+^ T cells compared with Rag1^KO^ colon (*P* = 0.0009, Figure 2G and Supplemental Figure 2A). Furthermore, the Rag1^KO^Tslpr^KO^ colon showed reduced Foxp3^+^CD4^+^ Treg frequency compared with the Rag1^KO^ colon (*P* = 0.0117, Figure 2H and Supplemental Figure 2B). In both groups of mice, colon-infiltrating CD4^+^ T cells differentiated into the effector memory phenotype (CD44^+^CD62L^–^) (Supplemental Figure 2, C–I). CD4^+^ T cells isolated from Rag1^KO^Tslpr^KO^ MLNs preferentially expressed IFN-γ (*P* = 0.0003, Figure 2I), while those in Rag1^KO^ MLNs exhibited higher expression of IL-17A compared with Rag1^KO^Tslpr^KO^ (*P* = 0.0009, Figure 2J). Likewise, a significantly higher percentage of colonic CD4^+^ T cells in Rag1^KO^Tslpr^KO^ mice expressed IFN-γ (*P* = 0.0103, Figure 2M and Supplemental Figure 2J), while the majority of CD4^+^ T cells in Rag1^KO^ colon expressed IL-17A (*P* = 0.0001, Figure 2N and Supplemental Figure 2J). Although IL-10–secreting CD4^+^ T cells showed no significant difference in frequency in the MLNs of the 2 groups (Figure 2K), significantly fewer colonic CD4^+^ T cells in Rag1^KO^Tslpr^KO^ mice expressed IL-10 compared with Rag1^KO^ controls (*P* = 0.0006, Figure 2O and Supplemental Figure 2J). No significant differences in IL-4^+^CD4^+^ T cell frequency were observed in the MLNs or colon of Rag1^KO^Tslpr^KO^ versus Rag1^KO^ mice (Figure 2, L and P, and Supplemental Figure 2J). Together, these results identify TSLP as a major regulator of homeostatic CD4^+^ T cell expansion in the adult colon, thereby preventing lethal colitis.

### Depletion of colonic microbiome blocks CD4^+^ T cell–induced colitis.

**Figure 3:**
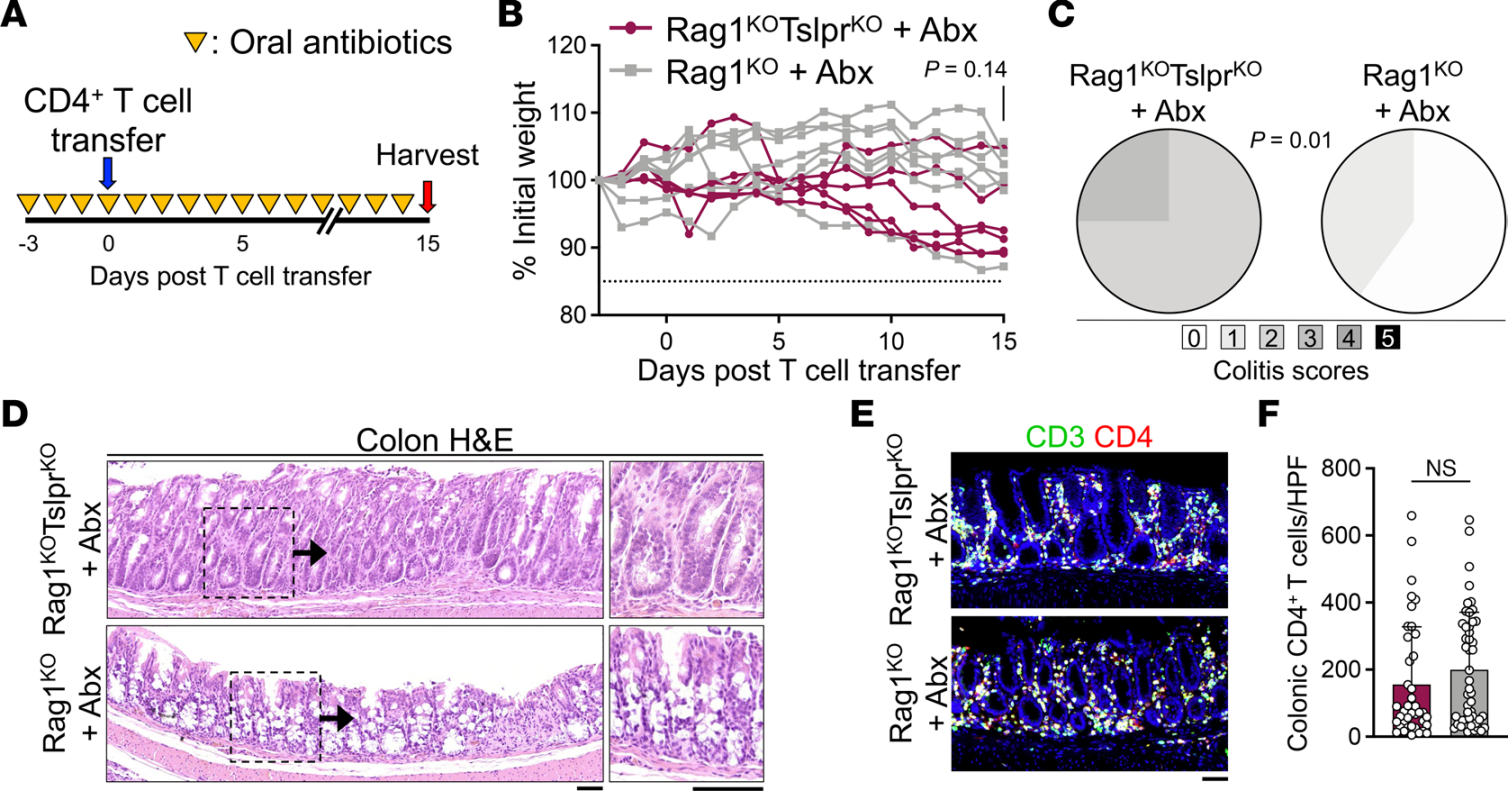
The colonic microbiome is necessary for CD4^+^ T cell expansion following adoptive transfer. (**A**) Schematic diagram of the microbiome-depleting antibiotic treatment regimen (Abx). Three days before adoptive CD4^+^ T cell transfer (blue arrow), mice were initiated on Abx daily (yellow triangles) and harvested 15 days after adoptive T cell transfer (red arrow). (**B**) Weight of Rag1^KO^Tslpr^KO^ (*n* = 6) and Rag1^KO^ (*n* = 7) treated with Abx and receiving adoptive CD4^+^ T cell transfer of 2 million purified naive WT CD4^+^CD25^–^ T cells. Unpaired, 2-tailed *t* test. (**C**) Colitis scoring of Rag1^KO^Tslpr^KO^ (*n* = 5) and Rag1^KO^ (*n* = 5) mice treated with Abx on day 15 after T cell transfer. Fisher’s exact test. (**D**) Representative colon H&E staining of Rag1^KO^Tslpr^KO^ and Rag1^KO^ mice treated with Abx on day 15 after T cell transfer. Insets show the high magnification of the mucosal inflammatory environment in the test and control groups. Note the mucosal hyperplasia in the Rag1^KO^Tslpr^KO^ sample, but the retention of goblet cells in both samples. Scale bars: 50 μm. (**E**) Representative CD3/CD4 staining of colons from Rag1^KO^Tslpr^KO^ and Rag1^KO^ mice treated with Abx on day 15 after T cell transfer. Scale bar: 50 μm. (**F**) CD4^+^ T cell counts in Rag1^KO^Tslpr^KO^ (*n* = 5) and Rag1^KO^ (*n* = 5) colons treated with Abx. CD4^+^CD3^+^ T cells in 10 randomly selected HPFs per colon were counted. Each dot represents 1 HPF. Experimental data were verified in a second independent experiment. Bar graph shows mean + SD. Unpaired, 2-tailed *t* test. NS, not significant.

Considering that the colonic microbiome is a major regulator of innate and adaptive immune response in the colon (25), we investigated its role in lethal colitis caused by incoming CD4^+^ T cells in Rag1^KO^Tslpr^KO^ mice. Rag1^KO^Tslpr^KO^ and Rag1^KO^ mice were treated with an oral antibiotic regimen consisting of vancomycin, neomycin, ampicillin, metronidazole, and gentamicin, which effectively eliminates the colonic microbiome (26). After antibiotic administration for 3 days, adoptive CD4^+^ T cell transfer was performed, and animals were harvested 15 days after T cell transfer (Figure 3A). No significant weight loss was observed in antibiotic-treated Rag1^KO^Tslpr^KO^ and Rag1^KO^ mice after CD4^+^ T cell transfer (*P* = 0.14, Figure 3B). As such, microbiome-depleting antibiotic treatment prevented lethal colitis in Rag1^KO^Tslpr^KO^ animals that received CD4^+^ T cell transfer. Antibiotic treatment markedly reduced the severity of CD4^+^ T cell–induced colitis in Rag1^KO^Tslpr^KO^ and Rag1^KO^ mice (Figure 3, C and D). Importantly, there was no difference in the numbers of colonic CD4^+^ T cells between Rag1^KO^Tslpr^KO^ and Rag1^KO^ mice (Figure 3, E and F). Moreover, no significant differences in CD45^+^, CD4^+^ T, Treg, or proliferating CD4^+^ T cell frequency were observed in the MLNs or colons of Rag1^KO^Tslpr^KO^ and Rag1^KO^ mice treated with antibiotics (Supplemental Figure 3, A–G). Likewise, no significant differences in IFN-γ, IL-17A, and IL-10 cytokine expression in CD4^+^ T cells were observed in the MLNs and colon of Rag1^KO^Tslpr^KO^ and Rag1^KO^ mice (Supplemental Figure 3, H-N). To examine whether microbiome depletion suppressed TSLP expression, WT colons were treated for 5 days with the same antibiotic regimen as above, which did not cause any significant change in colonic TSLP expression (Supplemental Figure 3O). These results demonstrate the essential role of the gut microbiome, likely as antigenic targets, in activating effector CD4^+^ T cells during their homeostatic expansion in Rag1^KO^ mice, which leads to lethal colitis in the absence of the host’s TSLP signaling.

### TSLP signaling in circulating cells prevents CD4^+^ T cell–induced lethal colitis in Rag1^KO^Tslpr^KO^ mice.

**Figure 4:**
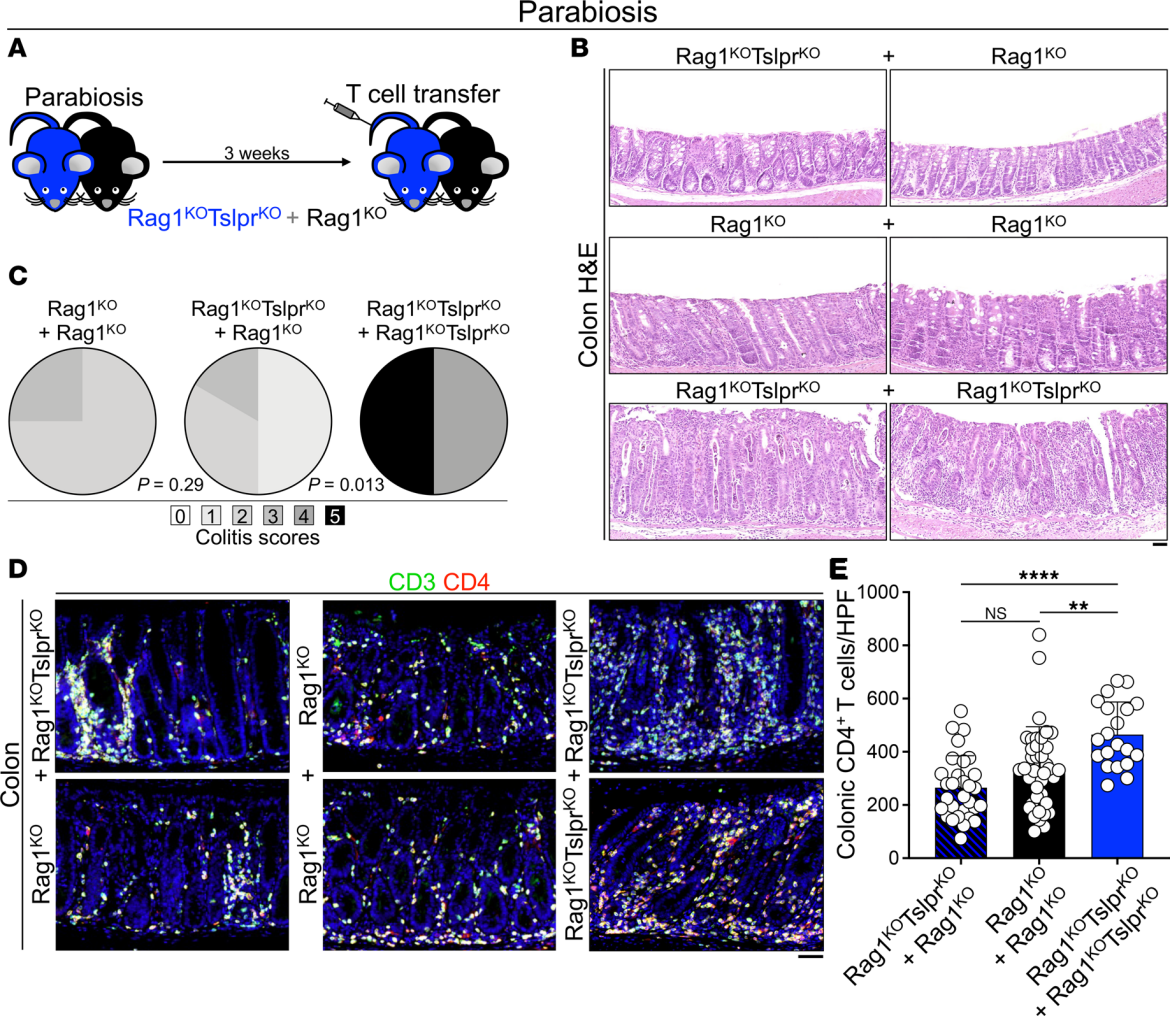
TSLP signaling in circulating cells suppresses CD4^+^ T cell expansion in the colon. (**A**) Diagram of parabiosis between Rag1^KO^Tslpr^KO^ and Rag1^KO^ mice. Three weeks after successful surgery, the Rag1^KO^Tslpr^KO^ mouse (if present in each pair) was given a total dose of 4 million purified naive WT CD4^+^CD25^–^ T cells i.v. (2 million/mouse). The animals were harvested at the moribund state or on day 15 after T cell transfer. (**B**) Representative colon H&E staining of Rag1^KO^Tslpr^KO^ + Rag1^KO^, Rag1^KO^ + Rag1^KO^, and Rag1^KO^Tslpr^KO^ + Rag1^KO^Tslpr^KO^ parabiotic pairs. Note the abundance of goblet cells in Rag1^KO^Tslpr^KO^ + Rag1^KO^ and Rag1^KO^ + Rag1^KO^ pairs, while Rag1^KO^Tslpr^KO^ + Rag1^KO^Tslpr^KO^ pairs have severe mucosal inflammation, crypt abscess formation, goblet cell loss, crypt distortion, and epithelial hyperplasia. Scale bars: 50 μm. (**C**) Colitis scoring of Rag1^KO^Tslpr^KO^ + Rag1^KO^ (*n* = 3 pairs), Rag1^KO^ + Rag1^KO^ (*n* = 2 pairs), and Rag1^KO^Tslpr^KO^ + Rag1^KO^Tslpr^KO^ (*n* = 2 pairs) parabiotic pairs. Fisher’s exact test. (**D**) Representative CD3/CD4 staining of colons from Rag1^KO^Tslpr^KO^ + Rag1^KO^, Rag1^KO^ + Rag1^KO^, and Rag1^KO^Tslpr^KO^ + Rag1^KO^Tslpr^KO^ parabiotic pairs. Scale bars: 50 μm. (**E**) CD4^+^ T cell counts in colon sections from Rag1^KO^Tslpr^KO^ + Rag1^KO^ (*n* = 3 pairs), Rag1^KO^ + Rag1^KO^ (*n* = 2 pairs), and Rag1^KO^Tslpr^KO^ + Rag1^KO^Tslpr^KO^ (*n* = 2 pairs) parabiotic pairs. CD3^+^CD4^+^ mucosal T cells were counted in 10 randomly selected HPFs per mouse at the end of the study. Each dot represents 1 HPF; bar graph shows mean + SD. **P < 0.01; ****P < 0.0001 by ordinary 1-way ANOVA. NS, not significant.

To determine whether TSLP signaling to the circulating versus colon-resident immune/stromal cells mediated the tolerogenic impact on transferred CD4^+^ T cells in the colon, we performed a parabiosis experiment in which Rag1^KO^Tslpr^KO^ mice were joined to Rag1^KO^ animals (Rag1^KO^Tslpr^KO^ + Rag1^KO^) before adoptive CD4^+^ T cell transfer (Figure 4A). PCR for the *Tslpr* WT allele in the parabiont’s blood shortly after the adoptive transfer of 4 million WT CD4^+^CD25^–^ T cells confirmed the connection of the circulation between the joint animals (Supplemental Figure 4A). Rag1^KO^Tslpr^KO^ parabiont in Rag1^KO^Tslpr^KO^ + Rag1^KO^ parabiotic pairs became protected from adoptive T cell transfer–induced severe colitis and exhibited similar levels of inflammation in the colon compared with Rag1^KO^ + Rag1^KO^ pairs, while the Rag1^KO^Tslpr^KO^ + Rag1^KO^Tslpr^KO^ pairs rapidly developed lethal colitis (Figure 4, B and C). The Rag1^KO^Tslpr^KO^ colon in Rag1^KO^Tslpr^KO^ + Rag1^KO^ pairs contained significantly fewer CD4^+^ T cells compared with the Rag1^KO^Tslpr^KO^ colon in Rag1^KO^Tslpr^KO^ + Rag1^KO^Tslpr^KO^ pairs (*P* < 0.0001) and was similar to CD4^+^ T cell counts in Rag1^KO^ colon in Rag1^KO^ + Rag1^KO^ pairs (*P* = 0.0969; Figure 4, D and E). These findings further confirmed that blood chimerism with Tslpr^WT^ animals protected the vulnerable Rag1^KO^Tslpr^KO^ hosts from severe colitis.

### TSLP signaling to DCs rescues CD4^+^ T cell–induced colitis.

**Figure 5:**
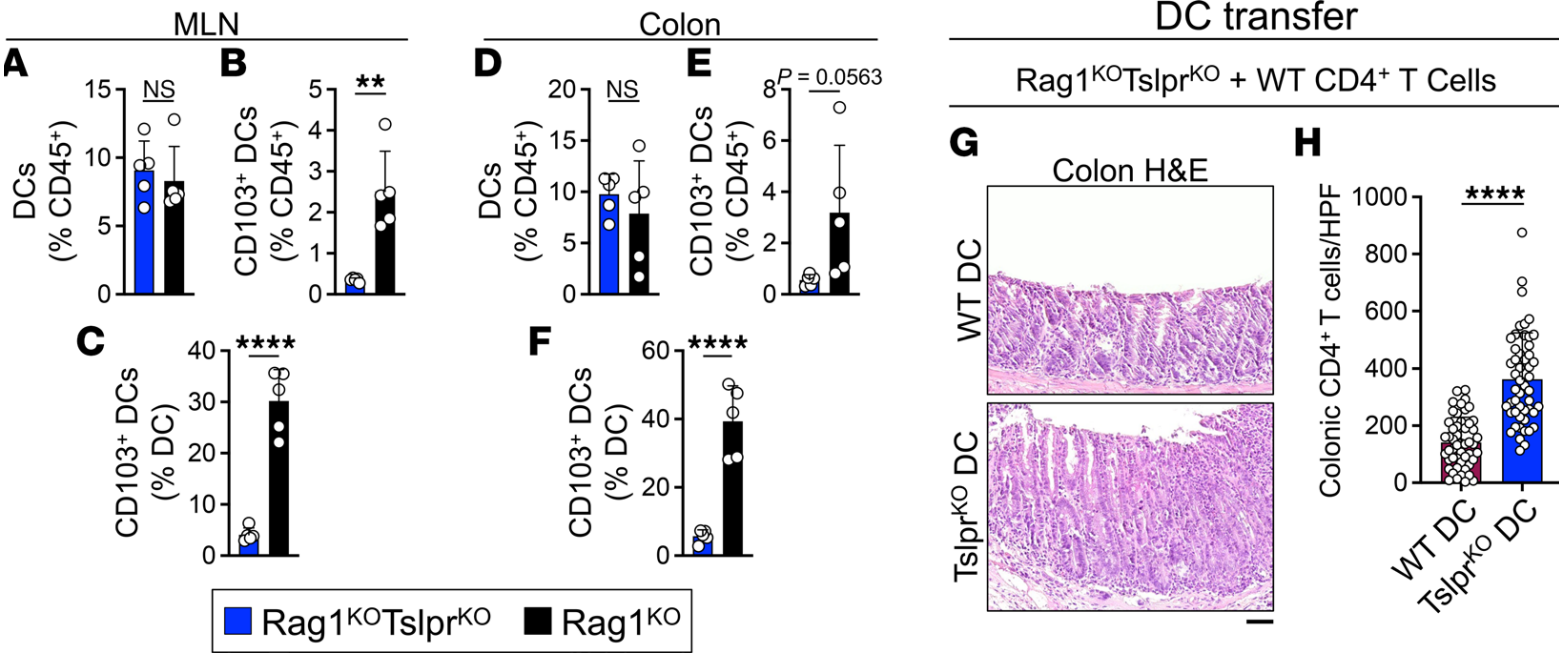
TSLP signaling to DCs suppresses CD4^+^ T cell expansion in the colon. (**A**–**C**) Flow cytometric quantification of CD11c^+^ myeloid DC frequency (**A**) and CD103^+^CD11c^+^ migratory DC frequency as a percentage of CD45^+^ leukocytes (**B**) versus CD11c^+^ myeloid DCs (**C**) in Rag1^KO^Tslpr^KO^ (*n* = 5) and Rag1^KO^ (*n* = 5) MLNs following adoptive transfer of naive WT CD4^+^CD25^–^ T cells. Rag1^KO^Tslpr^KO^ tissues were obtained at the moribund state and Rag1^KO^ samples were harvested on day 15 after T cell transfer. Each dot represents 1 mouse. (**D**–**F**) Flow cytometric quantification of CD11c^+^ myeloid DC frequency (**D**) and CD103^+^CD11c^+^ migratory DC frequency as a percentage of CD45^+^ leukocytes (**E**) versus CD11c^+^ myeloid DCs (**F**) in Rag1^KO^Tslpr^KO^ (*n* = 5) and Rag1^KO^ (*n* = 5) colon following adoptive transfer of naive WT CD4^+^CD25^–^ T cells. Rag1^KO^Tslpr^KO^ tissues were obtained at the moribund state and Rag1^KO^ samples were harvested on day 15 after T cell transfer. Each dot represents 1 mouse. (**G**) Representative H&E staining of colons from Rag1^KO^Tslpr^KO^ mice that received WT or Tslpr^KO^ DC transfer 1 day before adoptive CD4^+^ T cell transfer. Note the loss of goblet cells, moderate inflammation, and crypt hyperplasia in the animals given Tslpr^KO^ DCs compared with those that received WT DCs before T cell transfer. Scale bars: 50 μm. (**H**) CD4^+^ T cell counts in Rag1^KO^Tslpr^KO^ colons after the transfer of WT (*n* = 5) versus Tslpr^KO^ (*n* = 5) DCs followed by an adoptive naive WT CD4^+^CD25^–^ T cell transfer. CD3^+^CD4^+^ colonic T cells were quantified in 10 randomly selected HPFs per mouse at the moribund state or on day 15 after T cell transfer. Each dot represents 1 HPF; bar graphs show mean + SD. **P < 0.01; ****P < 0.0001 by unpaired, 2-tailed *t* test. NS, not significant.

Rag1^KO^Tslpr^KO^ and Rag1^KO^ animals that received WT CD4^+^ T cells were analyzed to identify candidate cell types for the protection phenotype observed. While there were no significant differences observed in overall CD11c^+^ myeloid DC numbers in the MLNs or colon of the 2 groups, CD103^+^ migratory DCs were preferentially found in the MLNs and colon of Rag1^KO^ compared with Rag1^KO^Tslpr^KO^ mice (Figure 5, A–F). Interestingly, even when the microbiome was depleted, CD103^+^ DCs showed higher frequency in the MLNs and colon of animals with intact TSLP signaling (*P* = 0.0104, *P* = 0.0252, respectively; Supplemental Figure 4, B–E). Antibiotic treatment reduced the number of DCs in the colon of Rag1^KO^ and Rag1^KO^Tslpr^KO^ mice (Supplemental Figure 4, F–I). To determine whether Tslpr^WT^ DCs were the cell type that protected Rag1^KO^ mice from adoptive T cell transfer–induced colitis, an adoptive transfer with either WT or Tslpr^KO^ CD11c^+^ DCs was performed 1 day before adoptive CD4^+^CD25^–^ T cell transfer into Rag1^KO^Tslpr^KO^ hosts. WT DCs significantly blunted colonic CD4^+^ T cell expansion and colitis in Rag1^KO^Tslpr^KO^ colon compared with Tslpr^KO^ DCs (*P* < 0.0001), indicating that Tslpr^WT^ DCs are sufficient to protect the host from severe colitis (Figure 5, G and H).

To determine the direct impact of TSLPR signaling in DCs on the regulation of CD4^+^ T cell response, bone marrow–derived DCs (BMDCs) from Tslpr^KO^ (test) versus WT (control) mice were generated and stimulated with recombinant TSLP (rTSLP) plus lipopolysaccharide (LPS) for 18 hours. Stimulated BMDCs were cocultured with CD4^+^ T cells from WT mice for 4 days (Supplemental Figure 5A). Tumor necrosis factor α (TNF-α) production by CD4^+^ T cells was significantly increased after coculture with Tslpr^KO^ compared with WT BMDCs (*P* = 0.0110; Supplemental Figure 5, B, C, and E). In contrast, Tslpr^KO^ BMDCs caused CD4^+^ T cells to produce significantly less IL-17A (*P* < 0.0001; Supplemental Figure 5, C and F) and IL-10 cytokines compared with WT BMDCs (*P* < 0.01; Supplemental Figure 5, C and G). There was no significant IL-4 induction observed in CD4^+^ T cells cocultured with Tslpr^KO^ or WT BMDCs (Supplemental Figure 5, C and H). Th1-driving cytokines, IL-12 and TNF-α, were increased in Tslpr^KO^ compared with WT BMDCs cocultured with CD4^+^ T cells (*P* < 0.0001, *P* = 0.0122, respectively; Supplemental Figure 5, B, D, I, and J). Importantly, IL-12 production by Tslpr^KO^ BMDCs was significantly increased compared with WT BMDCs upon rTSLP plus LPS stimulation and before coculture with T cells (*P* < 0.01, Supplemental Figure 5K). These findings indicate that TSLPR signaling in DCs induces tolerance during CD4^+^ T cell proliferation.

### Tslpr^KO^ mice show defect in establishing T cell tolerance in the colon.

**Figure 6:**
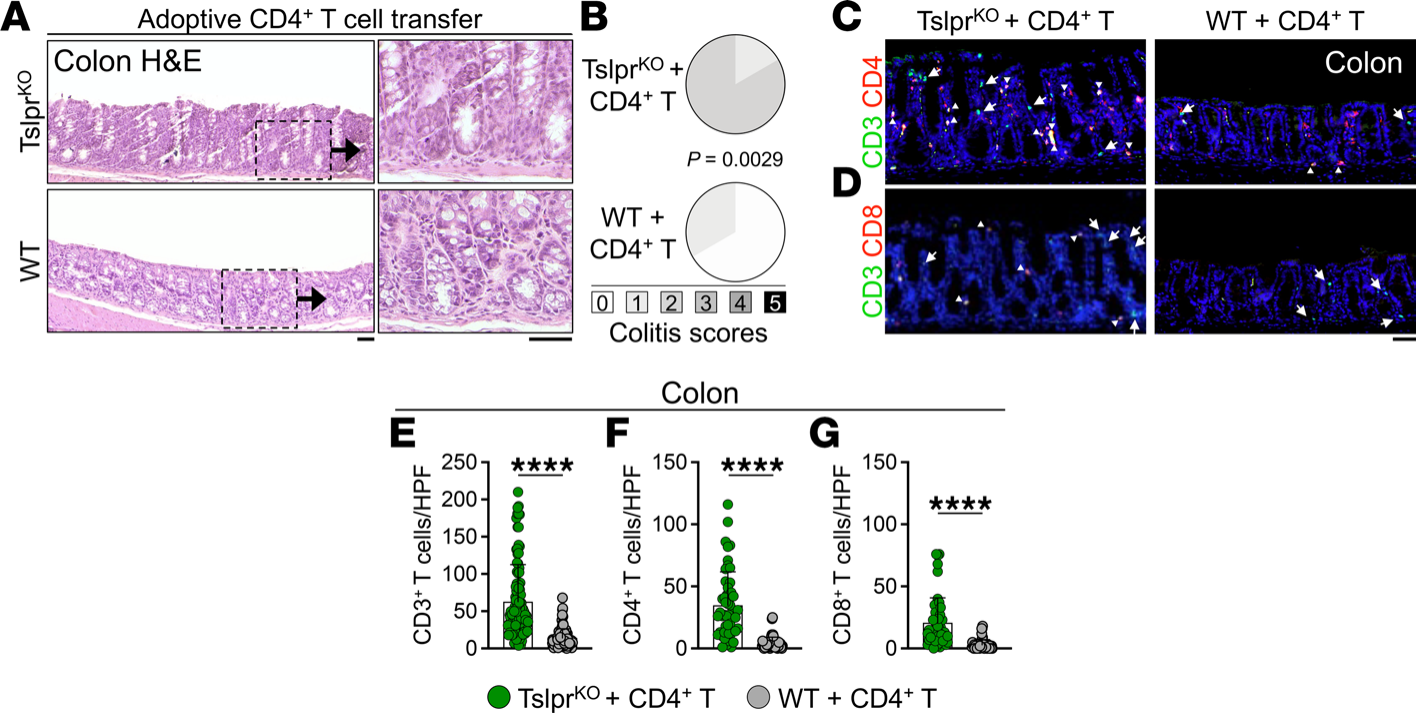
TSLP signaling protects against colitis after sublethal radiation and CD4^+^ T cell transfer in WT mice. (**A**) Representative H&E staining of colons from Tslpr^KO^ and WT colon on day 15 after adoptive CD4^+^ T cell transfer. Mice were sublethally irradiated and received an adoptive transfer of naive WT CD4^+^CD25^–^ T cells. Insets highlight the increased lymphocyte-dominated inflammatory environment present in the Tslpr^KO^ compared with the WT colon. Note the relative loss of goblet cells and increased epithelial crowding in the Tslpr^KO^ colon. Scale bars: 50 μm. (**B**) Colitis scoring of Tslpr^KO^ (*n* = 6) and WT (*n* = 6) on day 15 after T cell transfer. Fisher’s exact test. (**C** and **D**) Representative CD3/CD4 (**C**) and CD3/CD8 staining (**D**) of Tslpr^KO^ and WT colon 15 days after adoptive CD4^+^ T cell transfer. CD3 single-positive cells are highlighted with arrows and double-positive cells are highlighted with arrowheads. Note the increased mucosal CD4^+^ T cell presence preferentially in Tslpr^KO^ compared with WT colon. Scale bar: 50 μm. (**E**–**G**) CD3^+^ T (**E**), CD4^+^ T (**F**), and CD8^+^ T cell (**G**) counts in Tslpr^KO^ (*n* = 5) and WT (*n* = 5) colon on day 15 after T cell transfer. CD3^+^, CD3^+^CD4^+^, and CD3^+^CD8^+^ mucosal T cells were quantified in 10 randomly selected HPFs per colon at the end of the study. Each dot represents 1 HPF; bar graphs show mean + SD. ****P < 0.0001 by unpaired, 2-tailed *t* test.

To further substantiate TSLP’s role in peripheral T cell tolerance in the colon, we examined CD4^+^ T cell transfer into mice with intact T and B cells (19, 22). Colon TSLP levels were maintained in Tslpr^KO^ compared with WT animals (Supplemental Figure 6A). Tslpr^KO^ and WT animals received sublethal irradiation followed by an adoptive WT CD4^+^CD25^–^ T cell transfer. Although mice did not experience any weight loss, Tslpr^KO^ mice exhibited increased levels of colonic inflammation 15 days after T cell transfer (*P* = 0.0029; Figure 6, A and B). Furthermore, significantly higher numbers of CD3^+^ T (*P* < 0.0001), CD4^+^ T (*P* < 0.0001), and CD8^+^ T cells (*P* < 0.0001) infiltrated Tslpr^KO^ compared with WT colon (Figure 6, C–G). No significant colitis was observed in Tslpr^KO^ and WT mice that were sublethally irradiated without a CD4^+^ T cell transfer (Supplemental Figure 6B).

### TSLP signaling contributes to resistance to ICB-induced colitis in mice.

**Figure 7:**
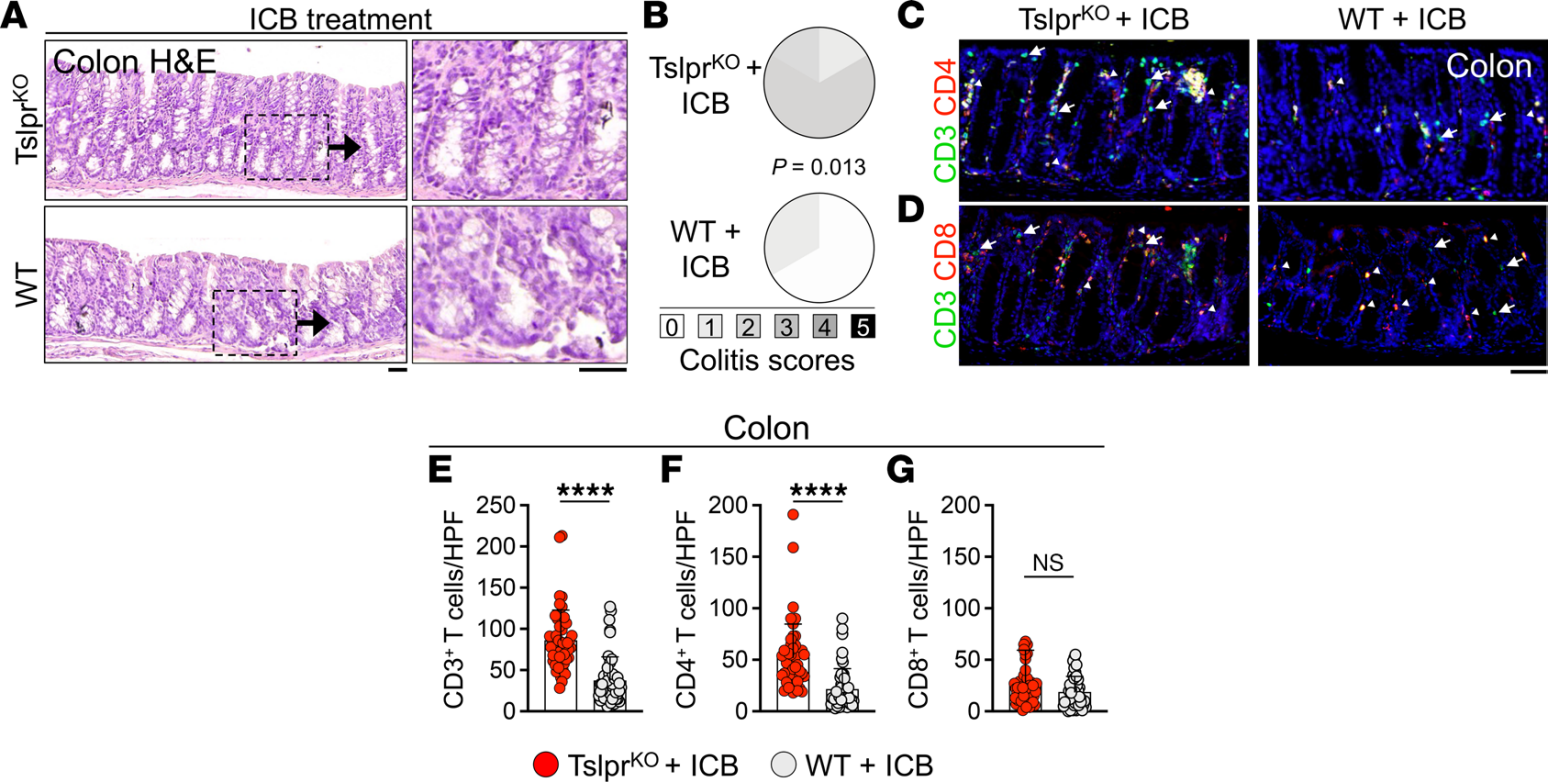
TSLP signaling protects the colon from immune checkpoint blockade–induced colitis. (**A**) Representative colon H&E staining of Tslpr^KO^ and WT mice treated intraperitoneally with 200 μg of anti–CTLA-4/anti–PD-1 immune checkpoint blockade (ICB) therapy every 3 days for 15 days and harvested on day 15 after therapy. Insets highlight the increased lymphocytic infiltrate in the Tslpr^KO^ compared with WT colon. Scale bars: 50 μm. (**B**) Colitis scoring of Tslpr^KO^ (*n* = 6) and WT (*n* = 6) on day 15 after therapy. Fisher’s exact test. (**C** and **D**) Representative CD3/CD4 (**C**) and CD3/CD8 staining (**D**) of Tslpr^KO^ and WT colon 15 days after ICB treatment. CD3 single-positive cells are highlighted with arrows and double-positive cells are highlighted with arrowheads. Note the increased mucosal CD4^+^ T cell presence preferentially in Tslpr^KO^ compared with WT colon. Scale bar: 50 μm. (**E**–**G**) CD3^+^ T (**E**), CD4^+^ T (**F**), and CD8^+^ T cell (**G**) count in Tslpr^KO^ (*n* = 5) and WT (*n* = 5) colon on day 15 after ICB therapy. CD3^+^, CD3^+^CD4^+^, and CD3^+^CD8^+^ mucosal T cells were quantified in 10 randomly selected HPFs per colon at the end of the study. Each dot represents 1 HPF; bar graphs show mean + SD. ****P < 0.0001 by unpaired, 2-tailed *t* test. NS, not significant.

Next, we examined the histology, colitis score, and immune cell infiltrates in the colon of Tslpr^KO^ versus WT mice at the baseline. The inflammatory environment of the colon was not significantly different between Tslpr^KO^ and WT mice (Supplemental Figure 6, C and D). The number of CD3^+^ T and CD8^+^ T cells did not significantly change in the colon of Tslpr^KO^ compared with WT mice (Supplemental Figure 6, E–G and I). However, CD4^+^ T cell counts were increased in Tslpr^KO^ compared with the WT colon at the baseline (*P* < 0.05, Supplemental Figure 6H). CD11c^+^ myeloid DC frequency did not differ in the MLNs or colon of Tslpr^KO^ and WT mice (Supplemental Figure 6, J–L). However, CD103^+^ migratory DCs were less frequent in Tslpr^KO^ compared with WT colon at the baseline (Supplemental Figure 6M). To determine whether TSLP-mediated peripheral tolerance complemented the immune checkpoints in mouse colon, we treated Tslpr^KO^ and WT animals with 200 μg anti–PD-1 plus 200 μg anti–CTLA-4 antibodies per mouse every 3 days for 15 days. Interestingly, ICB therapy led to increased inflammation (Figure 7, A and B) and significantly higher numbers of CD3^+^ T (*P* < 0.0001) and CD4^+^ T (*P* < 0.0001) but not CD8^+^ T cells in Tslpr^KO^ compared with WT colon (Figure 7, C–G). Taken together, these results indicate that TSLP serves as a homeostatic cytokine in the colon that cooperates with immune checkpoints in the establishment of peripheral T tolerance against the gut microbiome.

## Discussion

The contributions of type 2 alarmins to the pathogenesis of atopy is well characterized; however, the homeostatic function of these cytokines has only recently become apparent. Our studies into the impact of baseline TSLP expression on homeostatic CD4^+^ T expansion in barrier sites have revealed that colonic TSLP signaling is essential for suppressing the overexpansion of T cells and lethal colitis in adult animals. Loss of TSLP signaling unleashes an inflammatory Th1-like response in adoptively transferred CD4^+^CD25^–^ T cells against the gut microbiome. When TSLP signaling is functional on DCs, naive CD4^+^ T cells differentiate into Foxp3^+^CD4^+^ Tregs and a higher percentage of these cells express IL-10 and IL-17A cytokines in the colon. Previous studies have shown higher expression of IL-17 among other inflammatory cytokines released by colonic T cells in Tslpr^KO^ mice, which may indicate a direct impact of TSLP on T cells to block IL-17 expression in a steady state (27). However, the reduction of IL-17 expression by adoptively transferred WT CD4^+^CD25^–^ T cells in Rag1^KO^Tslpr^KO^ mice in our study suggests an indirect effect of TSLP on promoting the generation of IL-17^+^ colonic CD4^+^ T cells during homeostatic T cell expansion. Lower expression of IL-17A by WT CD4^+^ T cells in coculture with TSLPR-deficient DCs supports a role for TSLP signaling in DCs in promoting the generation of IL-17^+^ colonic CD4^+^ T cells. We found that the inflammatory cytokine IFN-γ is suppressed by TSLP signaling during homeostatic T cell expansion. This is consistent with previous studies demonstrating the reduction in inflammatory Th1 cell response after TSLP induction, which prevents colitis (3, 27). In the parabiosis model, circulating immune cells travel from the Tslpr^WT^ parabiont into the Tslpr^KO^ partner and confer protection from severe colitis. Furthermore, TSLP signaling on DCs is sufficient to restore tolerance among incoming CD4^+^ T cells in the colon. In the DC/T cell coculture system, Tslpr^KO^ DCs express high levels of IL-12 and activate CD4^+^ T cells to produce higher levels of TNF-α and lower levels of IL-17A and IL-10. Interestingly, loss of TSLP signaling in immunocompetent mice treated with ICB therapy leads to colitis, which is a common immune adverse event observed in cancer patients treated with ICB (28). Our findings identify TSLP as a major contributor to the homeostasis of the normal colon by engaging a DC-to–T cell communication to tolerize CD4^+^ T cells against healthy gut microbiome in adult animals.

Our findings demonstrate that barrier organs actively contribute to immune tolerance against the commensal microbiome. By releasing TSLP, the colon engages DCs and promotes the homeostasis of expanding T cells in the colon microenvironment. These results extend the role of TSLP from a pathogenic signal in atopy to a tolerogenic signal in barrier organs. TSLP has been shown to regulate inflammation in the colon by inducing a Th2 response to fight parasite infection (3, 15). In addition, TSLP induction in the colon contributes to mucosal healing after an inflammatory insult (16, 17). Previous work has shown that *Tslp* expression in the healthy colon is induced by the gut microbiome (17, 27); however, in our studies, we found TSLP protein levels to remain unchanged following microbiome-depleting antibiotic treatment. Our results indicate a link between TSLP and DCs in tolerizing the incoming CD4^+^ T cells against gut microbes in adult mice. TSLP can also directly induce colonic Tregs (27). Furthermore, the induction of TSLP expression in the skin induces systemic Treg generation through DC activation (29). Hassall’s corpuscles in the thymus express TSLP, which induces Tregs through DC activation (30). Our finding on the role of TSLP in engaging DCs to promote CD4^+^ T cell tolerance in the colon is consistent with the broader role of DCs in peripheral T cell tolerance in a healthy colon (31). In particular, CD103^+^ DCs are major contributors to the colonic homeostasis (32). Thus, our work provides a mechanism by which TSLP regulates the function of DCs in supporting tissue homeostasis.

The implications of our findings extend from the fundamental understanding of immune tolerance to the clinical manifestation of its break in patients. Considering that ICB therapy commonly leads to immune adverse events like dermatitis, colitis, and pneumonitis (28), understanding how TSLP and other alarmin cytokines regulate immune response to ICB therapy at barrier sites will have major therapeutic implications. Several groups have attempted to model ICB-induced colitis in mice without a clear phenotype. Our results suggest that blocking TSLP signaling may be necessary to predispose the mouse colon to ICB-induced colitis. Considering the reported similarities between immunopathogenesis of ICB-induced colitis and inflammatory bowel disease (IBD) (33–35), our findings on TSLP in the colon may provide an explanation for the observed link between the epithelium and IBD pathogenesis (36). TSLP expression is found to be reduced in patients with ulcerative colitis compared with controls, and TSLP level is inversely correlated with disease severity, which may suggest that TSLP is a protective signal in this disease (37). In patients with Crohn’s disease, TSLP is found to be downregulated compared with controls (38), further implicating TSLP as playing a pivotal role in suppressing inflammation in the colon. Thus, novel therapeutics targeting TSLP in the colon may prove effective for the treatment and prevention of IBD and ICB-induced colitis.

In summary, we show that TSLP acts as a tolerogenic cytokine in the colon by regulating the communication between DCs and CD4^+^ T cells. The implications of our findings extend beyond colitis and atopy and cast alarmins in a new light by identifying them as crucial mediators of immune cell homeostasis upon exposure to inflammatory stimuli at barrier organs. Rather than simply sounding the alarm, alarmin cytokines serve as fundamental inputs for microbiome-epithelium-immune system interactions in health and disease. Future studies into the homeostatic functions of alarmins are warranted to fully capture their therapeutic potential.

## Methods

### Mice.

All mice were given food and water ad libitum and housed under specific pathogen–free conditions with a 12-hour light/dark cycle in the animal facility at the Massachusetts General Hospital (MGH) consistent with animal care regulations. C57BL/6 *Rag1^–/–^* mice were purchased from the Jackson Laboratory. C57BL/6 Ly5.1 (B6/Ly5.1/Cr) mice were purchased from Charles River Lab (strain code: 564). C57BL/6 Tslpr^KO^ (a gift of Warren Leonard, NIH, Bethesda, Maryland, USA) and Rag1^KO^Tslpr^KO^ mice were bred in our facility to obtain mice to use in our studies. The test and control mice were cohoused for at least 2 weeks before experimentation to ensure similar microbiota composition. All the mice were maintained on the C57BL/6 background. Age-matched female mice were used as recipients in all adoptive transfer experiments.

### Tissue harvesting.

Mice were anesthetized using 200 μL of ketamine/xylazine. Following euthanasia, their organs, including the colon, small intestine, trachea, lung, and skin were collected for analysis. The mice’s back skin was shaved, excised, and fixed in 4% paraformaldehyde (PFA, Sigma-Aldrich). The colon was excised from the cecum to the rectum, flushed with phosphate-buffered saline (PBS) to remove feces, and opened longitudinally. Using a razor blade, approximately two-thirds of the colon was cut longitudinally and rolled for 4% PFA fixation or flash-frozen in OCT embedding medium (Thermo Fisher Scientific, catalog 23-730-571). The remaining one-third was used for RNA, protein, and flow cytometry analysis. A similar procedure was performed for small intestine specimens. The lung was filled with 1 mL 4% PFA by a syringe and excised from the most proximal part of the trachea. The trachea was later cut at the distal end and separated from the lung after fixation.

### Histology and immunofluorescence.

The barrier organs, including skin, colon, small intestine, trachea, and lung were harvested and fixed in 4% PFA and maintained at 4°C overnight. Tissues were washed with PBS and dehydrated in ethanol. The samples were processed and embedded in paraffin. Sections (5 μm) of paraffin-embedded tissues were cut and stained with hematoxylin and eosin (H&E). A part of the colon tissue was embedded in OCT, snap-frozen in liquid nitrogen, and stored at –80°C. For immunofluorescent staining, colon slides were rehydrated and permeabilized in 0.2% Triton X-100 for 5 minutes. The slides were then heated in antigen unmasking solution (Vector Laboratories, catalog H-3300) at high pressure in a Cuisinart pressure cooker for 20 minutes. Slides were then washed with 0.1% Tween 20 (Sigma-Aldrich, catalog P1379) 3 times for 5 minutes each in 1× Dulbecco’s PBS. Then, the sections were blocked in blocking buffer containing 1% bovine serum albumin (Thermo Fisher Scientific) and 5% goat serum (MilliporeSigma) for 1 hour and stained with anti-mouse primary antibodies (Supplemental Table 1) overnight at 4°C followed by fluorochrome-conjugated secondary antibodies and DAPI nuclear stain (Thermo Fisher Scientific). Slides were then mounted using 2–3 drops of mounting media (ProLong Gold Antifade reagent, Invitrogen). The NanoZoomer s60 digital scanner (Hamamatsu Corp.) was used to scan the slides, and high-resolution images were obtained by a Zeiss Axio Observer Z1 and analyzed using the Zeiss ZEN Image Processing software. The cell population was quantified within ×200-magnified high-power fields (HPFs) by the HALO Image Analysis Platform (Indica Labs).

### Colon H&E scoring.

Colon scoring was defined using 4 specified histology criteria as described previously and analyzed by a trained operator in a blind manner (39). These parameters included the severity, extent of tissue involvement, epithelial changes, and mucosal architecture. Based on these characteristics, the colon inflammation scored from 0 to 5. A score of 0 implied a noninflamed colon, while a score of 1 referred to minimal severity, only mucosal involvement with minimal epithelial hyperplasia and without mucosal architecture change. A score of 2 was defined as mild severity, mucosal and some submucosal involvement with mild hyperplasia and minimal goblet cell loss with or without erosions without mucosal architecture changes. A score of 3 represented moderate severity, mucosal and submucosal involvement with moderate hyperplasia, with or without few crypt abscesses and moderate goblet cell loss with or without erosions, without mucosal architecture changes. A score of 4 implied marked severity, mucosal and submucosal with marked hyperplasia, with or without several crypt abscesses and erosions, with or without irregular crypts or crypt loss and ulcerations. A score of 5 referred to marked severity, transmural involvement with marked hyperplasia, with or without multiple crypt abscesses, with or without irregular crypts or crypt loss with or without ulceration (Supplemental Table 2).

### Flow cytometry.

A portion of the distal colon (1/6) was mechanically dissociated. Colon and MLNs were incubated with collagenase IV (Worthington Biochemical) for 60 and 30 minutes, respectively, in a shaker at 37°C. After 60 minutes, the colon tissues were dissected for the second time, mashed, and filtered through a 70 μm cell strainer in a digestion buffer consisting of 1 mL RPMI 1640 medium (Gibco) and collagenase IV to obtain a single-cell suspension. The cells were centrifuged at 1200*g* for 5 minutes. For ex vivo experiments, the cultured cells were stained for live/dead cells using the Zombie NIR Fixable Viability kit (BioLegend) for 20 minutes to assess the cells’ viability. For in vivo and ex vivo studies, the cells were stained with cell surface antibodies (Supplemental Table 1) for 30 minutes. T cell cytokine stimulation was performed on a portion of the cells using a stimulation cocktail consisting of 50 ng/mL ionomycin (calcium salt, Sigma-Aldrich) and 500 ng/mL phorbol 12-myristate 13-acetate (PMA) (≥99% pure by TLC, Sigma-Aldrich) in 994 μL of R10 media consisting of fetal calf serum (Life Technologies), pen/strep/glutamine (Life Technologies), 2-meraptoethanol (Life Technologies), and RPMI 1640 by adding 10 μL of this cocktail to 990 μL of R10. After 1 hour in the 37°C incubator, 1 μL of brefeldin A (BioLegend) was added and incubated at 37°C for another 3 hours. The cells were spun down and stained with surface markers (Supplemental Table 1). Next, cells were fixed and permeabilized by a True-Nuclear Transcription Factor Buffer Set (BioLegend). After fixation and permeabilization, the cells were stained with anti-Foxp3 and anti-Ki67 intracellular antibodies and anti–IFN-γ, anti–IL-17A, anti–IL-10, anti–IL-4, anti–TNF-α, and anti–IL-12/23p40 intracellular antibodies for cytokine profiling at 4°C overnight. Stained cells were assayed using a Fortessa LSRII flow cytometer (BD Biosciences) and data were analyzed using FlowJo software (Tree Star).

### Adoptive T cell transfer.

Spleens were isolated from CD45.1^+^ WT donor mice on day –1. The cells were processed after 30 minutes of incubation at 37°C in 10 μL collagenase IV and 1 mL RPMI 1640 to obtain a single-cell suspension. WT CD4^+^CD25^–^ T cells were enriched using a MojoSort Mouse CD4 T Cell Isolation Kit (BioLegend), and then stained with anti-CD3ε, anti-CD4, anti-CD8α, anti-CD19, anti-CD25, and anti-NK1.1 monoclonal antibodies for cell sorting (Supplemental Table 1). WT CD4^+^CD25^–^ T cells were sorted by the SONY SH800 sorter (Sony Biotechnologies). Sorted CD3^+^CD4^+^CD8^–^CD19^–^CD25^–^NK1.1^–^ T cells (2 × 10^6^) were transferred into Rag1^KO^Tslpr^KO^ or Rag1^KO^ recipient mice by intravenous (i.v.) injection under isoflurane anesthesia. Mice were tracked for weight every day from day 0 until day 15. The mice were harvested when they lost 15% of their day 0 weight or reached day 15.

### Adoptive DC transfer.

Spleen and lymph nodes were isolated from CD45.1^+^ WT or Tslpr^KO^ donor mice and processed to obtain a single-cell suspensions. CD11c^+^ DCs were enriched using CD11c Microbeads UltraPure mouse (Miltenyi Biotec) and then stained with anti-CD45, anti-CD3ε, anti-CD8α, anti-CD19, anti-CD11c, and anti-MHCII antibodies for cell sorting (Supplemental Table 1). The cells were counted and 2 × 10^6^ CD3^–^CD4^–^CD19^–^CD45^+^CD11c^+^MHCII^+^ DCs were transferred i.v. into Rag1^KO^Tslpr^KO^ mice. The next day, CD3^+^CD4^+^CD8^–^CD19^–^CD25^–^NK1.1^–^ T cells from the spleen of CD45.1^+^ WT donor mice were isolated and transferred i.v. into recipient mice. The mice’s weight was measured daily until day 15 after T cell transfer. Mice were harvested when they lost 15% of their weight or reached day 15.

### Antibiotic therapy.

Starting 3 days before adoptive T cell transfer, mice were placed on a high dose gavage treatment of 200 μL antibiotic, consisting of 1 mg/mL ampicillin (anhydrous, 96.0%–102.0%, Sigma-Aldrich), gentamicin sulphate (Sigma-Aldrich), metronidazole (Metronidazole BioXtra, Sigma-Aldrich), neomycin (trisulfate salt hydrate, Sigma-Aldrich), and 0.5 mg/mL vancomycin (vancomycin hydrochloride, *Streptomyces* *orientalis*, potency ≥900 μg/mg, Sigma-Aldrich) diluted in PBS. On day 0, mice received 2 × 10^6^ WT CD4^+^CD3^+^CD25^–^ T cells i.v. and were monitored for change in weight over 15 days.

### Parabiosis.

The pairs of parabiotic mice were housed together for at least 2 weeks before the surgery to ensure pair compatibility. Three pairs of C57BL/6 female Rag1^KO^Tslpr^KO^ + Rag1^KO^Tslpr^KO^, Rag1^KO^ + Rag1^KO^, and Rag1^KO^Tslpr^KO^ + Rag1^KO^ mice were connected surgically. The pairs were matched for weight and age. The animals were anesthetized with 3%–4% isoflurane and the surgery was performed on a heating pad. The mice’s skin was shaved on the lateral side, where the incisions would be made and sterilized with Betadine and 70% ethanol. A longitudinal incision along one side of each mouse was made. Elbow and knee joints from each animal were attached by passing a 4-0 surgical suture with a double surgical knot. The skin of the animals was sealed with a continuous absorbable 5-0 Vicryl suture, starting ventrally from the elbow toward the knee and continuing dorsally, ending with a double surgical knot. Carprofen (0.1 mg; Rimadyl, Zoetis, catalog 141-199) was injected subcutaneously to minimize pain and repeated for 2 days after surgery. The mice were closely monitored every day for a sign of stress and pain. Sutures were removed on day 7 to 14. On day 28, the animals received WT CD4^+^ T cell transfer and were tracked for another 15 days. The mice were euthanized on day 15 and their colon, MLNs, and small intestine were collected for analysis.

### ICB therapy.

Mice received intraperitoneal injections of 200 μg (~10 mg/kg) anti–mouse PD-1 (clone 29F.1A12, BioXCell, catalog BE2073) and 200 μg anti–mouse CTLA-4 (clone 9D9, BioXCell, catalog BE0164) blocking antibodies or 400 μg IgG isotopes (1:1 mixture of rat IgG [Sigma-Aldrich, catalog no. i4313] and mouse IgG [Southern Biotech, catalog 0107-01]) as control every 3 days. The mice were monitored for change in weight every 3 days for 15 days.

### Protein isolation.

Protein isolation was performed by placing the harvested organs in tissue lysis buffer consisting of 4% protease inhibitor (Thermo Fisher Scientific) in PBS plus 0.1% v/v Tween 20 (Sigma-Aldrich). Tissue was homogenized using a TissueLyser II (Qiagen) at a frequency of 30/s for 5 minutes. The homogenized samples were transferred into new tubes and frozen in liquid nitrogen for 1 minute, and then thawed in a 56°C water bath for 1 minute and sonicated for 30 seconds. The samples were centrifuged at 13,226*g* for 10 minutes at 4°C. The supernatants were transferred to a new tube and stored at –80°C until analysis.

### ELISA.

The expression of TSLP, IL-25, and IL-33 in barrier organs and IL-12/23(p40) protein levels in BMDC culture media were measured by ELISA, using LEGEND MAX TSLP, IL-33, IL-25 (IL-17E), and IL-12/23(p40) ELISA Kits following the manufacturer’s instructions (BioLegend). The total protein was measured by BCA Protein Assay Kit (Thermo Fisher Scientific) and the same amount of total protein was used for the analysis across the samples. The cytokine concentrations were measured at 450 nm using a Synergy Neo2 (BioTek) and were calculated by a Gen5 Microplate Reader and Imager Software (BioTek) with a 5-parameter logistic curve.

### DC/T cell coculture.

Bone marrow was harvested from the tibia of Tslpr^KO^ versus WT mice on the C57BL/6 background. Bone marrow cells were plated in nontreated cell culture dishes with 10 mL R10 and stimulated with 20 ng/mL recombinant murine granulocyte-macrophage colony-stimulating factor (GM-CSF, PeproTech, catalog 315-03) for 8 days to generate BMDCs. Media were changed on day 5. On day 8, BMDCs were harvested and quantified and plated in 24-well plates in R10 at 1 × 10^5^/mL concentration and supplemented with 10 ng/mL LPS (from *E*. *coli* O55:B5, Sigma-Aldrich, catalog L6529-1MG) and 10 ng/mL rTSLP (eBioscience, 34-8498-82) for 18 hours. BMDC supernatant was harvested after 18 hours for ELISA. BDMCs were cocultured at 1:1 with 1 × 10^5^/mL WT CD4^+^CD25^–^ T cells supplemented with 10 ng/mL LPS and 10 ng/mL rTSLP for 4 days. On day 4, the cells were stimulated with PMA, ionomycin, and brefeldin A for the final 4 hours and analyzed by flow cytometry.

### Statistics.

All the graphs were created and statistical analyses were performed using GraphPad Prism 9. A log-rank test was used to compare the animals’ survival. Fisher’s exact test was used to compare colitis grades. A 2-tailed, unpaired *t* test was used to compare animals’ weight, cytokine levels, and immune cell quantifications. The ordinary 1-way ANOVA test was used for multiple comparisons when comparing more than 2 groups. A *P* value of less than 0.05 was considered significant.

### Study approval.

Animal studies were approved by Massachusetts General Hospital Institutional Animal Care and Use Committee (IACUC, protocol 2015N000089).

## Author contributions

JLM, MA, and SD conceived and designed the experiments. JLM, MA, and KED performed the experiments and analyzed the data. JLM, MA, and SD interpreted the data and wrote the manuscript.

## Supplementary Material

Supplemental data

## Version 1 07/10/2023

Electronic publication
