# Supplementary material for: TSLP/dendritic cell axis promotes CD4+ T cell tolerance to the gut microbiome
Source: JCI Insight. 2023 Jul 10;8(13):e160690. doi: 10.1172/jci.insight.160690 (PMC10371333; doi:10.1172/jci.insight.160690)
Supplement: Supplemental data [file jciinsight-8-160690-s134.pdf]

TSLP/Dendritic cell axis promotes $CD4^+$ T cell tolerance to the gut microbiome

Jonathan L. Messerschmidt[1], Marjan Azin[1], Kaitlin E. Dempsey[1] and Shadmehr Demehri[1]*

SUPPLEMENTAL FIGURES AND TABLES

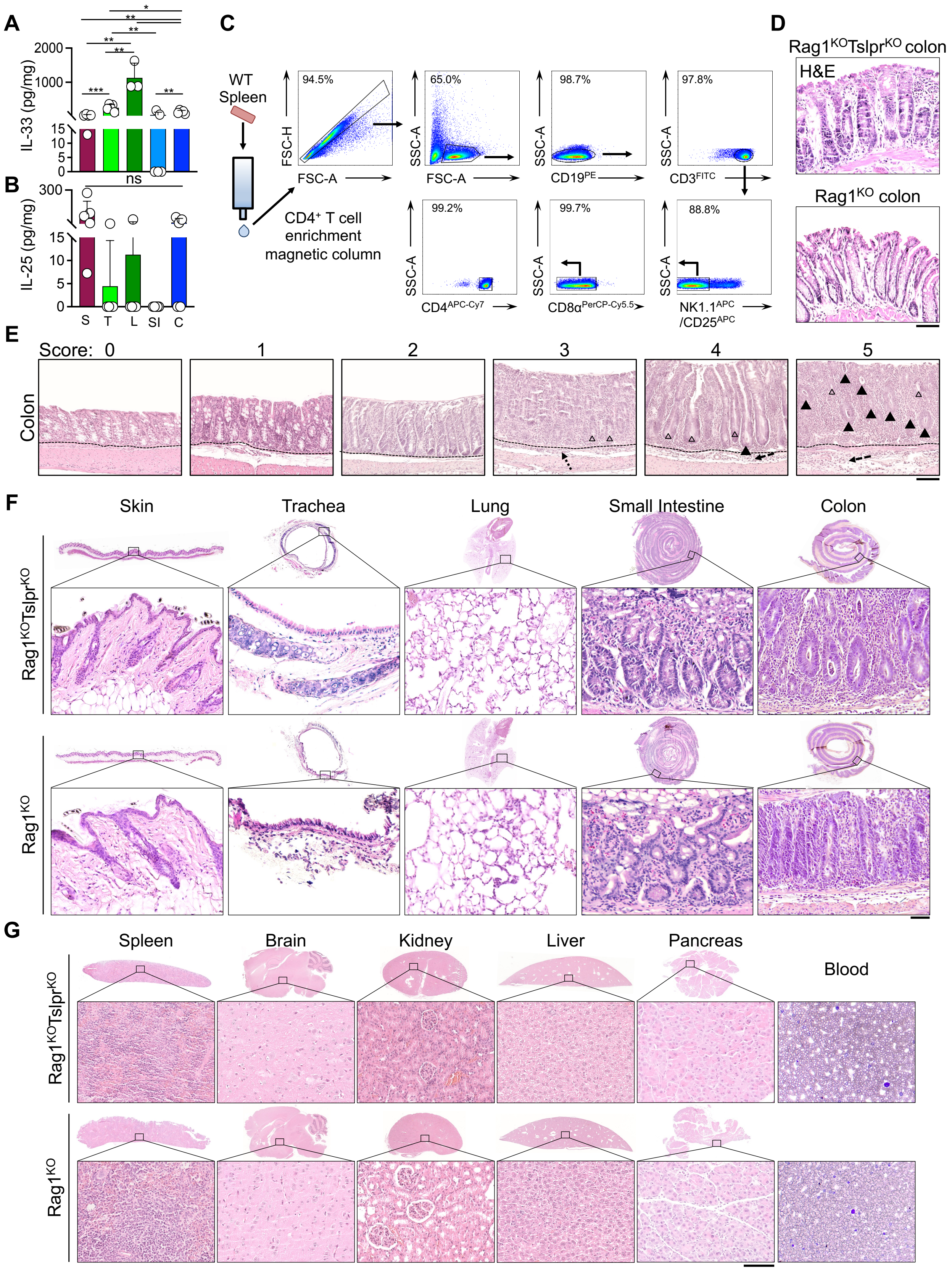
A
B
C
D
E
F
G
IL-33 (pg/mg)
IL-25 (pg/mg)
ns
S
T
L
SI
C
WT Spleen
CD4+ T cell enrichment magnetic column
94.5%
65.0%
98.7%
97.8%
99.2%
99.7%
88.8%
FSC-H
FSC-A
SSC-A
CD19PE
CD3FITC
CD4APC-Cy7
CD8αPerCP-Cy5.5
NK1.1APC /CD25APC
Rag1KOTslprKO colon
H&E
Rag1KO colon
Score: 0
1
2
3
4
5
Colon
Skin
Trachea
Lung
Small Intestine
Colon
Rag1KOTslprKO
Rag1KO
Spleen
Brain
Kidney
Liver
Pancreas
Blood

Supplemental Figure 1. **Epithelium-derived alarmins levels in normal barrier organs, $CD4^+$ T cells sorting and its impact on vital organs after adoptive transfer.**

(**A** and **B**) IL-33 (A) and IL-25 (B) protein concentration in WT mouse (n = 5) skin (S), trachea (T), lung (L), small intestine (SI), and colon (C). Bar graphs show mean + sd, Ordinary one-way ANOVA, ns: not significant, *$P < 0.05$, **$P < 0.01$, ***$P < 0.001$.

(**C**) Representative $CD4^+$ T cell sorting strategy. WT splenocytes were passed over a $CD4^+$ T cell enrichment column before pooling samples and FACS sorting for $CD19^-CD3^+CD25^-NK1.1^-CD8^-$ $CD4^+$ T cells using the outlines gating strategy. The percentage of cells within the gate is shown on each flow dot plot.

(**D**) Representative H&E stains of $Rag1^{KO}Tslpr^{KO}$ cells and $Rag1^{KO}$ colon at the baseline. Scale bars, 50 µm.

(**E**) Representative H&E-stained adoptive T cell transfer-induced colitis histology and associated score. This scoring system was used as a rubric to grade specimens in the study, as described previously (1). Note the retention of goblet cells in the mucosal tissue in scores 0-2 with mild increases in crypt height indicating crypt hyperplasia as the scores increase. Overall cellularity increases through the first three scores as well. A score of 3 is notable for the invasion of inflammatory cells beyond the muscularis mucosa (dotted line) and into the submucosal tissue (dotted arrow). A score of 4 includes severe lack of goblet cell presence (open triangles), while a score of 5 is notable for the formation of several crypt abscesses (solid triangles) as well as severe crypt distortion and absorption by inflammatory tissue. Crypt hyperplasia and submucosal infiltration continue throughout scores 3-5 as well. Scale bar, 50 µm.

(**F**) Representative H&E stains of barrier organs from $Rag1^{KO}Tslpr^{KO}$ and $Rag1^{KO}$ after adoptive transfer of WT $CD4^+CD25^-$ T cells. Scale bar, 50 µm.

(**G**) Representative H&E stains of several vital organs and Giemsa stains of peripheral blood smears from $Rag1^{KO}Tslpr^{KO}$ and $Rag1^{KO}$ after adoptive transfer of WT $CD4^+CD25^-$ T cells. Scale bar, 50 µm.

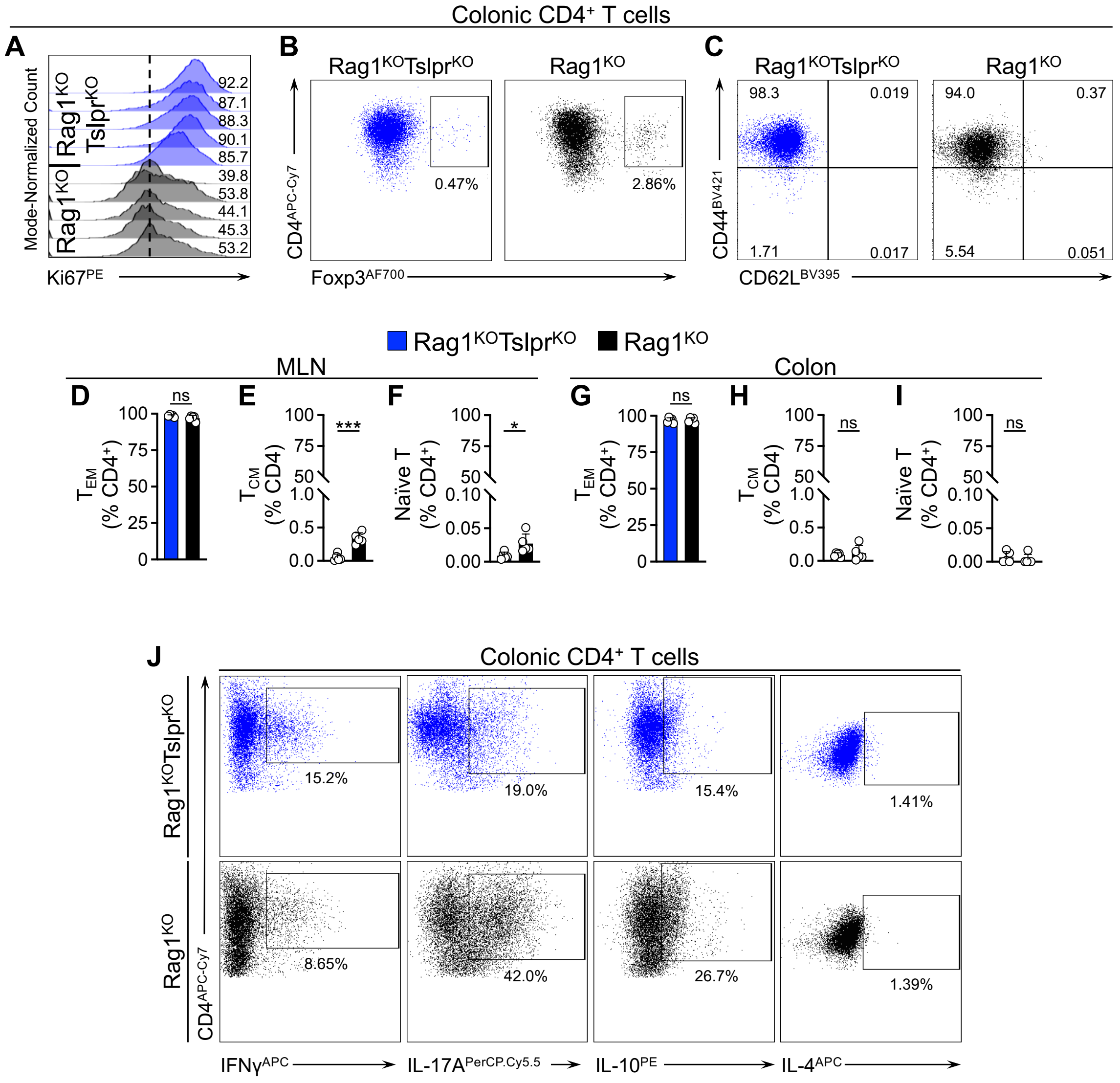

Supplemental Figure 2. **CD4$^+$ T cells status in recipient Rag1^{KO}Tslpr^{KO} and Rag1^{KO} mice.**

(**A**) Representative flow histograms of Ki67 expression in the colonic CD4$^+$ T cells from Rag1^{KO}Tslpr^{KO} (blue) and Rag1^{KO} (black) at the end of the study. The percentage of Ki67$^+$CD4$^+$ T cells is shown on the histograms.

(**B**) Representative flow cytometric dot plots of Foxp3$^+$CD4$^+$ T$_{reg}$ in Rag1^{KO}Tslpr^{KO} (blue) and Rag1^{KO} (black) colon.

(**C**) Representative flow cytometric dot plots of CD44 and CD62L expression on CD4$^+$ T cells adoptively transferred into Rag1^{KO}Tslpr^{KO} (blue) and Rag1^{KO} (black) colon.

(**D**-**F**) Quantification of $CD4^+$ T cell phenotypes: T_{EM}, effector memory T cell ($CD44^+CD62L^-$, D), T_{CM}, central memory T cell ($CD44^+CD62L^+$, E) and Naïve T cell ($CD44^-CD62L^+$, F) in $Rag1^{KO}Tslpr^{KO}$ and $Rag1^{KO}$ MLN at the end of the study.

(**G**-**I**) Quantification of $CD4^+$ T_{EM} (G), T_{CM} (H) and Naïve T cell (I) in $Rag1^{KO}Tslpr^{KO}$ and $Rag1^{KO}$ colon at the end of the study.

(**J**) Representative flow cytometric dot plots of $IFN\gamma^+$, IL-17A$^+$, IL-10$^{+,}$ and IL-4$^+$ $CD4^+$ T cells in $Rag1^{KO}Tslpr^{KO}$ (blue) and $Rag1^{KO}$ (black) colon.

The percent cells in each gate are shown on the flow plots. Each dot represents one mouse, bar graphs show mean + sd, unpaired *t*-test; ns, not significant, **P* < 0.05, ****P* < 0.001.

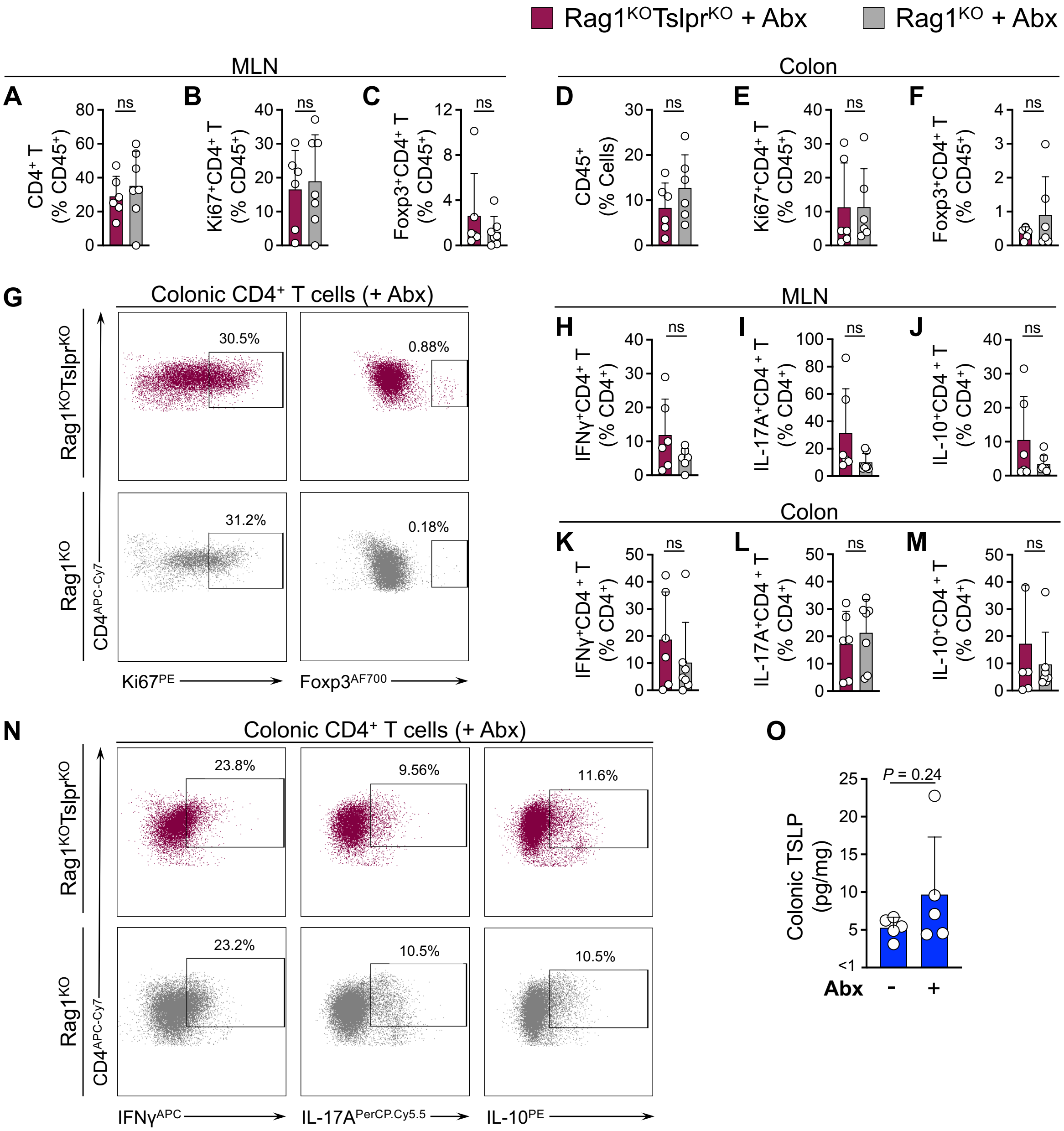

Supplemental Figure 3. **The microbiome does not alter colonic TSLP expression in adult mice**.

(**A**-**C**) Flow cytometric quantification of $CD4^+$ T (A), $Ki67^+CD4^+$ T (B) and $Foxp3^+CD4^+$ T_{reg} (C) frequency from mesenteric lymph nodes (MLN) of $Rag1^{KO}Tslpr^{KO}$ (n = 5) and $Rag1^{KO}$ (n = 5) mice. Experimental data were verified in a second independent experiment.

(**D**-**F**) Flow cytometric quantification of $CD4^+$ T (D), $Ki67^+CD4^+$ T (E) and $Foxp3^+CD4^+$ T_{reg} (F) frequency in $Rag1^{KO}Tslpr^{KO}$ (n = 5) and $Rag1^{KO}$ (n = 5) colons. Experimental data were verified in a second independent experiment.

(**G**) Representative flow cytometric dot plots of $Ki67^+CD4^+$ T and $Foxp3^+CD4^+$ T_{reg} in $Rag1^{KO}Tslpr^{KO}$ and $Rag1^{KO}$ colons. The percent cells in each gate are shown on the flow plots.

(**H**-**J**) Flow cytometric quantification of $IFN\gamma^+$ (H), IL-$17A^+$ (I) and IL-10^+ (J) $CD4^+$ T cell frequency from MLN of $Rag1^{KO}Tslpr^{KO}$ (n = 5) and $Rag1^{KO}$ (n = 5) mice. Experimental data were verified in a second independent experiment.

(**K**-**M**) Flow cytometric quantification of $IFN\gamma^+$ (K), IL-$17A^+$ (L) and IL-10^+ (M) $CD4^+$ T cell frequency in $Rag1^{KO}Tslpr^{KO}$ (n = 5) and $Rag1^{KO}$ (n = 5) colons. Experimental data were verified in a second independent experiment.

(**N**) Representative flow cytometric dot plots of $IFN\gamma^+$, IL-$17A^+$ and IL-10^+ $CD4^+$ T cells in $Rag1^{KO}Tslpr^{KO}$ and $Rag1^{KO}$ colons. The percent cells in each gate are shown on the flow plots.

(**O**) TSLP expression in the colon of WT adult mice with or without microbiome-depleting antibiotic treatment (Abx).

Single-cell suspensions from MLN and colon were stimulated for 4 h with PMA/ionomycin + Brefeldin A and analyzed for cytokine production. Each dot represents one mouse, bar graphs show mean + sd, unpaired *t*-test, ns: not significant.

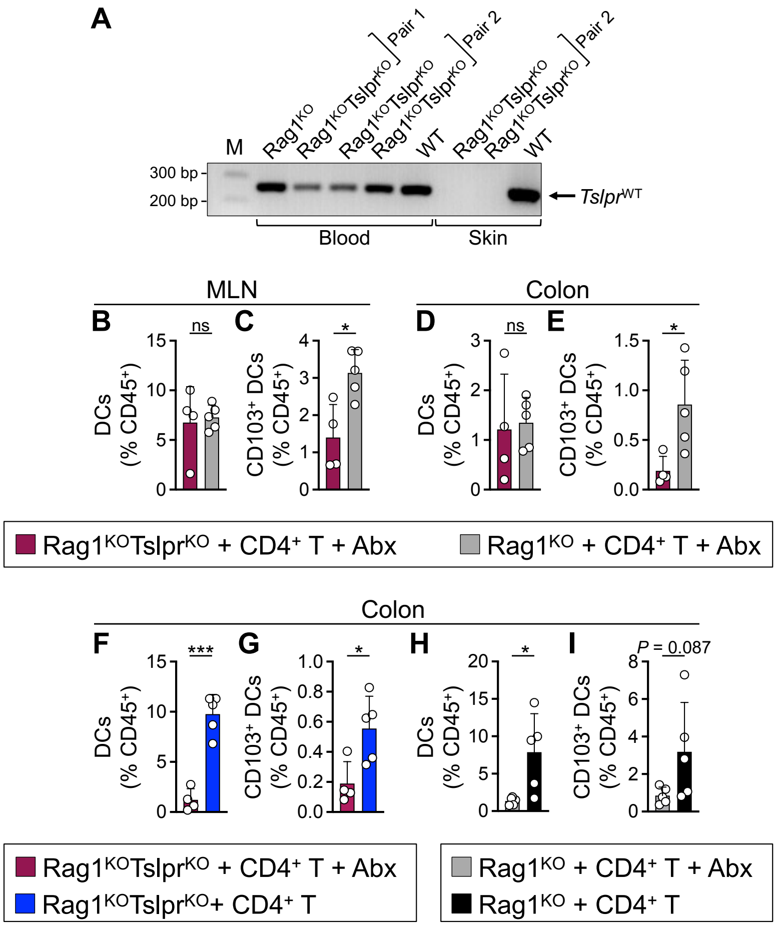

Supplemental Figure 4. **Confirmation of circulatory chimerism in parabiotic animals and DC status in antibiotic-treated mice.**

(**A**) Tslpr wild-type allele (Tslpr^{WT}) PCR detection in blood and skin samples from Rag1^{KO}Tslpr^{KO} and Rag1^{KO} parabionts following adoptive transfer of WT CD4^{+} T cells into one animal in each parabiotic pair. WT mouse blood and skin are shown as the positive control. Note that Rag1^{KO}Tslpr^{KO} + Rag1^{KO}Tslpr^{KO} pair (#2) has Tslpr^{WT} cells in the blood of both parabionts but not skin. M, marker.

(**B** and **C**) Flow cytometric quantification of CD11c^{+} myeloid DC (B) and CD103^{+}CD11c^{+} migratory DC (C) frequency in Rag1^{KO}Tslpr^{KO} (n = 4) and Rag1^{KO} (n = 5) MLN following antibiotics treatment and adoptive transfer of naïve WT CD4^{+}CD25^{-} T cells.

(**D** and **E**) Flow cytometric quantification of $CD11c^+$ myeloid DC (D) and $CD103^+CD11c^+$ migratory DC (E) frequency in $Rag1^{KO}Tslpr^{KO}$ (n = 4) and $Rag1^{KO}$ (n = 5) colon following antibiotics treatment and adoptive transfer of naïve WT $CD4^+CD25^-$ T cells. $Rag1^{KO}Tslpr^{KO}$ and $Rag1^{KO}$ tissues were harvested at day 15 post-T cell transfer.

(**F** and **G**) Flow cytometric quantification of $CD11c^+$ myeloid DC (F) and $CD103^+CD11c^+$ migratory DC (G) frequency in $Rag1^{KO}Tslpr^{KO}$ + $CD4^+$ T cell and antibiotics (n = 4) versus $Rag1^{KO}Tslpr^{KO}$ + $CD4^+$ T cell (n = 5) colon.

(**H** and **I**) Flow cytometric quantification of $CD11c^+$ myeloid DC (H) and $CD103^+CD11c^+$ migratory DC (**I**) frequency in $Rag1^{KO}$ + $CD4^+$ T cell and antibiotics (n = 5) versus $Rag1^{KO}$ + $CD4^+$ T cell (n = 5) colon.

Each dot represents one mouse, bar graphs show mean + sd, unpaired *t*-test, ns: not significant, *P < 0.05, ***P = 0.0001.

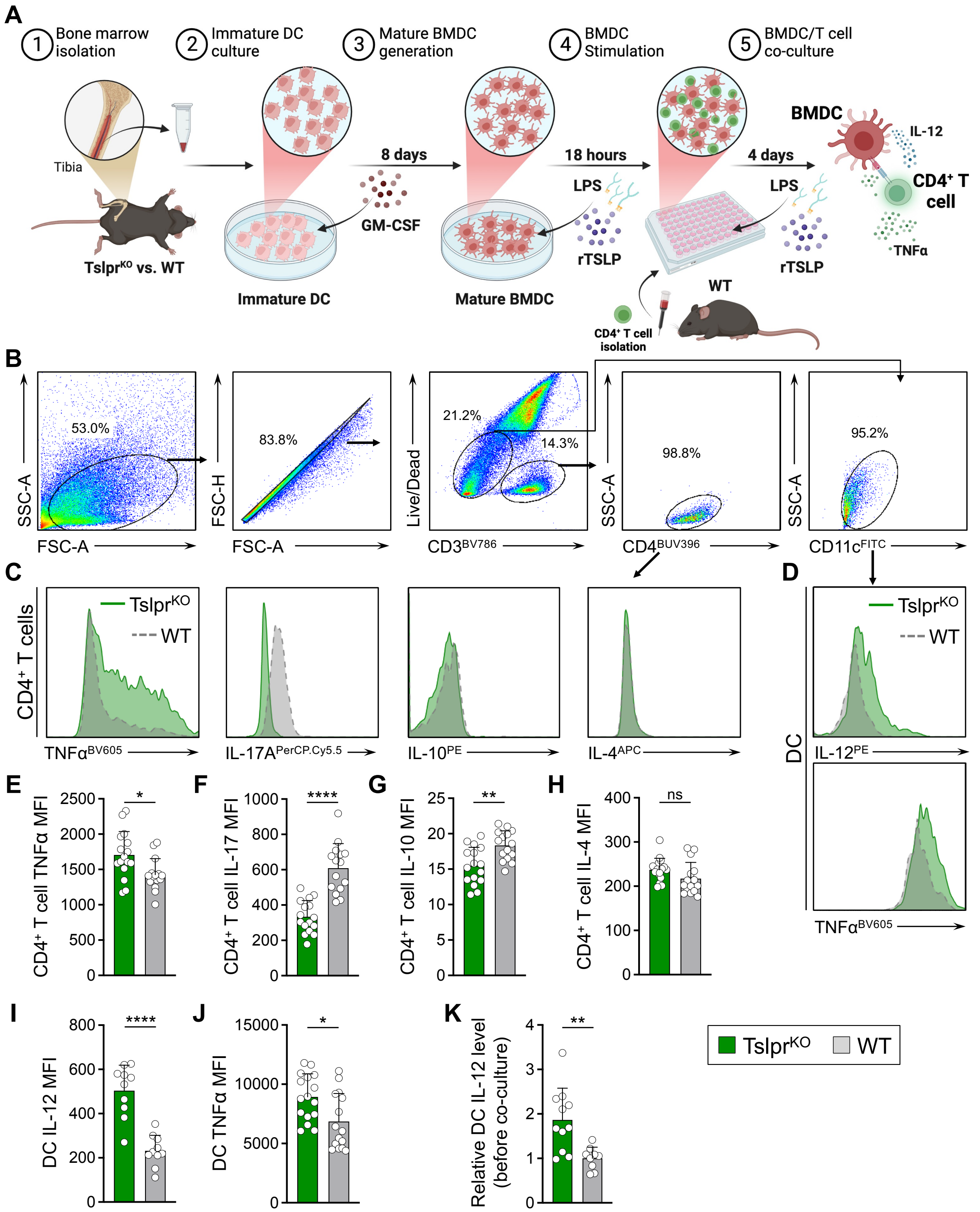

Supplemental Figure 5. Loss of TSLPR signaling in DCs increases IL-12 expression by DCs and proinflammatory cytokine expression by CD4⁺ T cells in a co-culture assay.

(**A**) Schematic diagram of BMDC/T cell co-culture assay in which Tslpr^{KO} versus WT BMDC is generated, stimulated and then co-cultured with CD4^{+} T cells.

(**B**) Flow gating strategy to assess BMDCs and $CD4^+$ T cells after BMDC/T cell co-culture.

(**C**) Representative flow histogram of TNFα, IL-17A, IL-10 and IL-4 expression in $CD4^+$ T cells after co-culture with $Tslpr^{KO}$ versus WT BMDCs.

(**D**) Representative flow histogram of IL-12 and TNFα expression in $Tslpr^{KO}$ versus WT BMDCs after co-culture with WT $CD4^+$ T cells.

(**E**-**H**) Quantification of $CD4^+$ T cell TNFα (E), IL-17A (F), IL-10 (G), and IL-4 (H) MFI after co-culture with $Tslpr^{KO}$ versus WT BMDCs.

(**I** and **J**) Quantification of $Tslpr^{KO}$ versus WT BMDC IL-12 (I) and TNFα (J) MFI after co-culture with WT $CD4^+$ T cells.

(**K**) Relative IL-12 levels in the media collected from $Tslpr^{KO}$ (n = 11) versus WT (n = 9) BMDCs after rTSLP plus LPS stimulation for 18 hours before co-culture with $CD4^+$ T cells. Data is combined from two independent experiments. Each dot represents media from a BMDC sample, bar graphs show mean + sd, unpaired *t*-test, *: $P < 0.01$, **: $P < 0.01$, ****: $P < 0.0001$, ns: not significant.

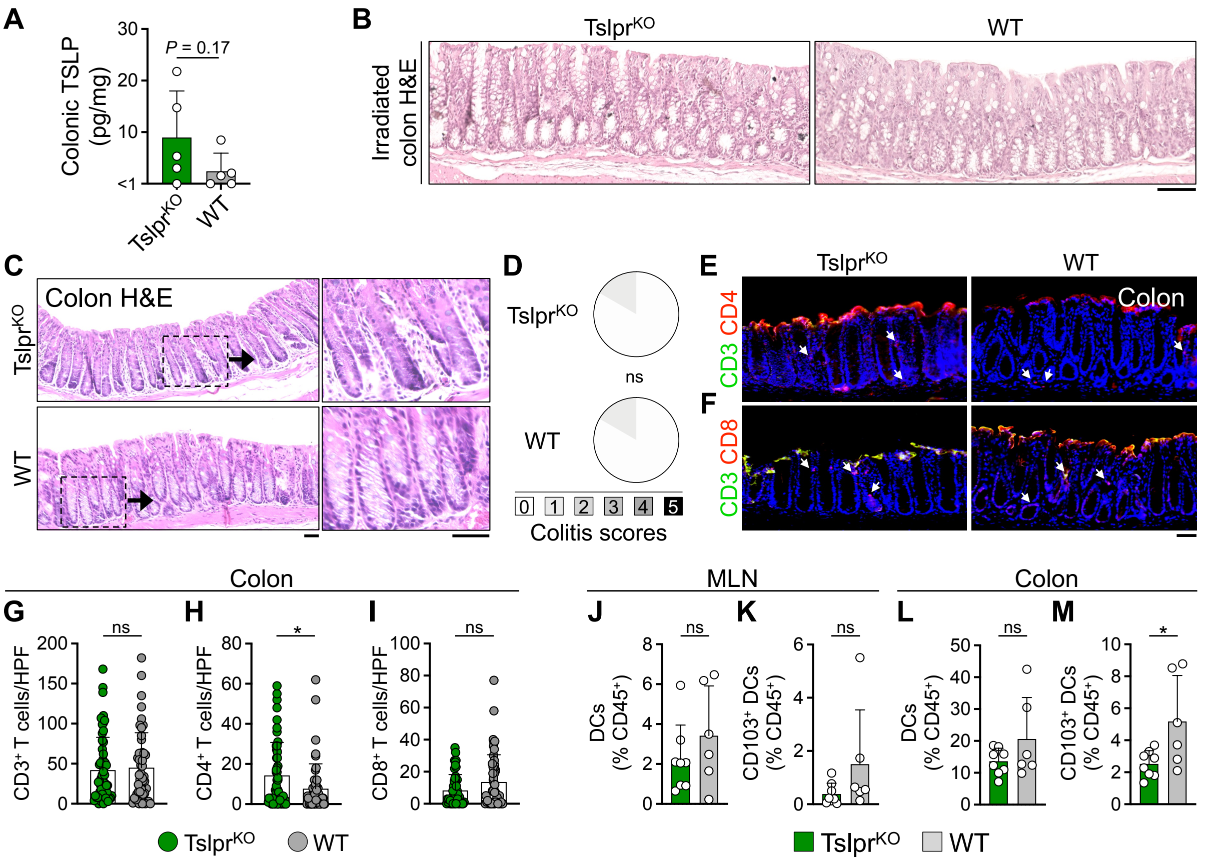

Supplemental Figure 6. **The inflammatory environment and T cell infiltrates in Tslpr^{KO} and WT colon at the baseline.**

(**A**) TSLP protein levels in Tslpr^{KO} and WT colon at baseline. Each dot represents one mouse.

(**B**) Representative H&E stains of sublethally irradiated Tslpr^{KO} (n = 5) and WT (n = 5) colon from two independent experiments at day 15 post-irradiation. Note the retention of goblet cells, the relative paucity of immune cells in a thin mucosal interstitium, normal crypt structure and depth. Scale bar, 50 µm.

(**C**) Representative H&E stains of colons from Tslpr^{KO} and WT colon at baseline. Scale bars, 50 µm.

(**D**) Colitis scoring of Tslpr^{KO} (n = 5) and WT (n = 5) at baseline. Fisher's exact test.

(**E-F**) Representative CD3/CD4- **(**E**)** and CD3/CD8-staining (F) of Tslpr^{KO} and WT colon at baseline. CD3$^+$CD4$^+$ and CD3$^+$CD8$^+$ cells are highlighted with white arrows. Scale bar, 50 µm.

(**G**-**I**) $CD3^+$ T (G), $CD4^+$ T (H) and $CD8^+$ T cell (I) quantification in $Tslpr^{KO}$ (n = 5) and WT (n= 5) colon at baseline. $CD3^+$, $CD3^+CD4^+$ and $CD3^+CD8^+$ mucosal T cells were quantified in 10 randomly selected HPF images per colon at the end of the study. Each dot represents one HPF image.

(**J**-**M**) Flow cytometric quantification of DC and $CD103^+$ DC frequency in MLN (J and K) and colon (L and M) of $Tslpr^{KO}$ (n = 8) versus WT (n = 6) mice. Each dot represents one mouse.

Bar graphs show mean + sd, unpaired *t*-test, *: *P* < 0.01, ns: not significant.

Supplemental Table 1. Antibodies used in the study.

Flow cytometry Antibodies	Conjugate	Clone	Manufacturer	Cat #	Isotype	RRID
CD3	BV785	17A2	BioLegend	100232	Rat IgG2b, κ	AB_2562554
CD3	FITC	145-2C11	BioLegend	100306	Armenian Hamster IgG	AB_312671
CD4	APC-Cy7	RM4-5	BioLegend	100526	Rat IgG2a, κ	AB_312727
CD8α	PE-Cy7	53-6.7	BioLegend	100722	Rat IgG2a, κ	AB_312761
CD8α	FITC	53-6.7	Biolegend	100706	Rat IgG2a, κ	AB_312745
CD8α	APC	53-6.7	Biolegend	100712	Rat IgG2a, κ	AB_312751
CD8α	APC-eF780	53-6.7	eBioscience	47-0081-82	Rat / IgG2a, kappa	AB_1272185
CD8α	PerCP-Cy5.5	53-6.7	BioLegend	100734	Rat IgG2a, κ	AB_2075238
CD19	PE	1D3/CD19	BioLegend	152408	Rat IgG2a, κ	AB_2629817
CD19	FITC	eBio1D3	eBioscience	11-0193-85	Rat / IgG2a, kappa	AB_657666
NK1.1	APC	PK136	BioLegend	108710	Mouse IgG2a, κ	AB_313397
NK1.1	FITC	PK136	BioLegend	108706	Mouse IgG2a, κ	AB_313393
CD45	PerCP-Cy5.5	30-F11	BioLegend	103131	Rat IgG2b, κ	AB_893344
CD45	BV421	30-F11	BioLegend	103133	Rat IgG2b, κ	AB_10899570
CD45	FITC	30-F11	BioLegend	103108	Rat IgG2b, κ	AB_312973
CD11c	APC-Cy7	N418	BioLegend	117324	Armenian Hamster IgG	AB_830649
CD11c	PE	N418	BioLegend	117308	Armenian Hamster IgG	AB_313777
CD103	Alexa Fluor 700	2E7	Invitrogen	56-1031-82	Armenian hamster / IgG	AB_2637111
CD11b	BUV395	M1/70	BD Bioscience	563553	Rat DA, also known as DA/HA IgG2b, κ	AB_2738276
MHCII	PE	M5/114.15.2	BD Bioscience	557000	Rat BN x LEW IgG2b, κ	AB_396546
MHCII	AF647	M5/114.15.2	BioLegend	107618	Rat IgG2b, κ	AB_493525
Foxp3	APC	FJK-16s	eBioscience	17-5773-82	Rat IgG2a, κ	AB_469457
IFNγ	Alexa Fluor 700	XMG1.2	BioLegend	505824	Rat IgG1, κ	AB_2561300
IL-17A	PerCP-Cy5.5	TC11-18H10.1	BioLegend	506919	Rat IgG1, κ	AB_961385
IL-10	PE	JES5-16E3	BioLegend	505007	Rat IgG2b, κ	AB_315361
IL-4	APC	11B11	BioLegend	504106	Rat IgG1, κ	AB_315320
IL-12	PE	C15.6	BioLegend	505204	Rat IgG1, κ	AB_315368
Ki67	Alexa Fluor 700	16A8	BioLegend	652419	Rat IgG2a, κ	AB_2564284
TNFα	BV605	MP6-XT22	BioLegend	506333	Rat IgG1, κ	AB_2562450
Zombie NIR	APC-Cy7	-	BioLegend	423105	-	-
**Immunofluorescence Antibodies**	**Conjugate**	**Clone**	**Manufacturer**	**Cat #**	**Isotype**	**RRID**
CD3	Purified	CD3-12	Abcam	Ab11089	Rat IgG1	AB_369097

CD4	Purified	EPR19514	Abcam	AB183685	Rabbit IgG	AB_2686917
CD8	Purified	D4W2Z	Cell Signaling Technologies	98941	Rabbit IgG	AB_2756376
TSLP	Purified	73AD11	Merck	GNE01.12F 3.B5.4011	Rat IgG2a	
Cytokeratin	Purified	AE1/AE3	Dako	M3515	IgG1 kappa	
Myeloperoxidase (MPO)	Purified	EPR20257	Abcam	ab208670	Rabbit IgG	
**Blocking Antibodies**	**Conjugate**	**Clone**	**Manufacturer**	**Cat #**	**Isotype**	**RRID**
PD1	Purified	29F.1A12	BioXCell	BE0273		
CTLA-4	Purified	9D9	BioXCell	BE0164		

Supplemental Table 2. Colon inflammation (colitis) scoring.

Minimal	Mucosa	Minimal hyperplasia		1
Mild	Mucosa, some submucosa	Mild hyperplasia, minimal goblet cell loss +/- erosions		2
Moderate	Mucosa and submucosa	Moderate hyperplasia +/- few crypt abscesses, moderate goblet cell loss +/- erosions		3
Marked	Mucosa and submucosa	Marked hyperplasia +/- several crypt abscesses and or erosions	+/- Irregular crypts or crypt loss +/- ulcerations	4
Marked	Transmural	Marked hyperplasia +/- multiple crypt abscesses	+/- Irregular crypts or crypt loss +/- ulcerations	5

Reference:

1. Erben U, Loddenkemper C, Doerfel K, Spieckermann S, Haller D, Heimesaat MM, et al. A guide to histomorphological evaluation of intestinal inflammation in mouse models. *Int J Clin Exp Pathol.* 2014;7(8):4557-76.
